# Resolving the Emission Transition Dipole Moments of Single Doubly Excited Seeded Nanorods via Heralded Defocused Imaging

**DOI:** 10.1021/acs.nanolett.3c00155

**Published:** 2023-06-08

**Authors:** Daniel Amgar, Gur Lubin, Gaoling Yang, Freddy T. Rabouw, Dan Oron

**Affiliations:** † Department of Physics of Complex Systems, Weizmann Institute of Science, Rehovot 76100, Israel; ‡ School of Optics and Photonics, Beijing Institute of Technology, Beijing 100081, China; § Debye Institute of Nanomaterials Science, Utrecht University, Princetonplein 1, 3584 CC Utrecht, The Netherlands; ∥ Department of Molecular Chemistry and Materials Science, Weizmann Institute of Science, Rehovot 76100, Israel

**Keywords:** SPAD arrays, single-particle spectroscopy, Heralded Spectroscopy, emission anisotropy, biexciton, emission transition dipole

## Abstract

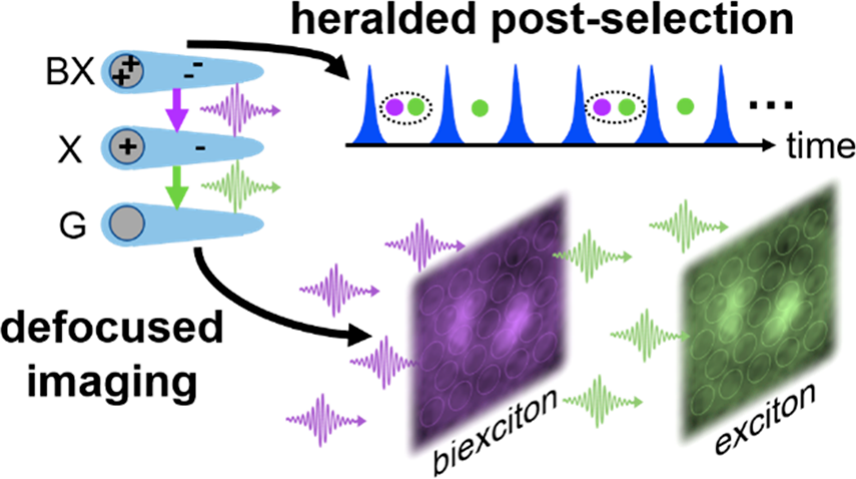

Semiconductor nanocrystal emission polarization is a crucial probe of nanocrystal physics and an essential factor for nanocrystal-based technologies. While the transition dipole moment for the lowest excited state to ground state transition is well characterized, the dipole moment of higher multiexcitonic transitions is inaccessible via most spectroscopy techniques. Here, we realize direct characterization of the doubly excited-state relaxation transition dipole by heralded defocused imaging. Defocused imaging maps the dipole emission pattern onto a fast single-photon avalanche diode detector array, allowing the postselection of photon pairs emitted from the biexciton–exciton emission cascade and resolving the differences in transition dipole moments. Type-I^1^/_2_ seeded nanorods exhibit higher anisotropy of the biexciton-to-exciton transition compared to the exciton-to-ground state transition. In contrast, type-II seeded nanorods display a reduction of biexciton emission anisotropy. These findings are rationalized in terms of an interplay between the transient dynamics of the refractive index and the excitonic fine structure.

In recent years, spectroscopy of single particles has evolved to provide a profound understanding of intrinsic properties of semiconductor nanocrystals (NCs). This is due to a combination of advances in synthesis and dramatic advances in single-photon detection systems. The improvements in synthesis protocols over the years yield photostable, tunable, and bright semiconductor nanocrystals (NCs), enabling deeper spectroscopic investigations at the single-particle level, as well as utilization in a plethora of applications. One of the most important features of NCs, as light emitters, is polarization of optical transitions, experimentally characterized by the dimensionality and orientation of their excitation and emission transition dipoles.^1^ Emission polarization from CdSe spherical microcrystals, which originates from their hexagonal lattice, was theoretically and experimentally reported in the early 1990s.^2,3^ Nanorod (NR) architectures, quantum-confined in two dimensions, have been shown to preferentially emit linearly polarized light due to a combination of the anisotropic lattice and dielectric effects due to the anisotropic shape. According to previous reports, the highly polarized NR emission indicates that the emissive transition has a one-dimensional (1D) dipole along the long axis of the rods.^4−6^ Several studies have used polarization microscopy to determine the three-dimensional (3D) orientation of single symmetric fluorophores, with the requirement to have a twofold degenerate transition dipole oriented isotropically in two dimensions.^7−10^ Alternatively, Jasny et al. and other groups have proposed the method of defocused imaging for finding the 3D orientation of a radiating dipole based on the analysis of light intensity distribution, supported by theoretical calculations.^11−16^ Notably, however, all of the above studies have characterized the transition dipole moment of the singly excited state relaxation, which typically accounts for most of the emission in photoluminescence experiments. The transition dipole moments of higher (multiple) excited states remain largely unexplored. Indeed, spectroscopic characterization of multiply excited states is difficult due to their low emission quantum yield and rapid decay dynamics.^17^ Yet, since multiply excited states play a crucial role in achieving optical gain and in the development of quantum light sources, developing a sophisticated, yet facile, approach to directly probe the dipole moment of higher-excited states at the single-particle level is required. Specifically, much attention has been directed toward spectroscopic characterization of the doubly excited or biexciton (BX) state.^18,19^ A BX is formed upon double excitation of a single NC and may decay to the ground state (GS) through a cascade process, emitting two consecutive photons. The first photon can be assigned to the BX-to-exciton (X) transition and the second photon to the X-to-GS transition, termed here BX and X photons, respectively. Recently, Heralded Spectroscopy has demonstrated unambiguous isolation of multiexciton emission through temporal photon correlations using single-photon avalanche diode (SPAD) detectors array.^20,21^ Isolating the BX state from other emissive states through photon correlations features significant advantages over ensemble detection schemes, such as transient absorption (TA) and time-resolved photoluminescence (PL), in which the interpretation of the results is more challenging due to a combination of inhomogeneous broadening and contributions from higher excited, often also charged, states.^22−24^ Crucially for this work, the heralded postselection of BX and X emission is a single-NC method rather than an ensemble technique and as such does not average over emitter orientations.

Here, we report the direct measurement of emission anisotropy of the BX state by coupling a defocused imaging setup to a two-dimensional (2D) SPAD array and applying heralded postselection of X and BX emission. This technique, dubbed Heralded Defocused Imaging, allows estimation of the difference between the emission anisotropy of the X and BX emission transition dipole. The experimental results show higher anisotropy of the BX-to-X transition over the X-to-GS transition for type-I^1^/_2_ (quasi-type-II) CdSe/CdS and the opposite trend for type-II ZnSe/CdS seeded NRs. We rationalize these findings by discussing possible competing contributions from both the transient dynamics of the refractive index and the excitonic fine structure.

To investigate the transition dipole moment orientation of the BX-to-X transition, we examine two nanoheterostructures that are known to emit partially polarized light from the X-to-GS transition. The first is CdSe/CdS seeded NRs and the second is ZnSe/CdS seeded NRs, denoted here as NR1 and NR2, respectively. Both heterostructures feature a quasi-spherical core (CdSe or ZnSe) within an elongated CdS rod shell. Figure 1a shows transmission electron microscopy (TEM) images of the nanocrystals investigated in this work. The band alignment differences between the two nanocrystal types, illustrated in Figure 1b, result in qualitatively different charge-carrier wave functions, making them interesting candidates for this comparison. NR1 features a charge carrier distribution characteristic of a type-I^1^/_2_ (also known as quasi-type-II) band alignment, where the hole is localized to the CdSe core and the electron is delocalized across the core and the rod. NR2 features a type-II band alignment, where the hole and the electron are separated to the ZnSe core and CdS rod, respectively.^25^ Notably, in both cases Coulomb attraction affects mostly the electron wave function distribution within the rod. The two seeded NR systems exhibit similar absorption profiles far from the band edge (λ < 450 nm, where absorption is dominated by the CdS rod) but different absorption profiles around the lowest excitonic peak, as shown in Figure 1c. The emission peaks of both systems are close to 600 nm. Further details of the NCs can be found in section S1 of the Supporting Information (SI).

**Figure 1:**
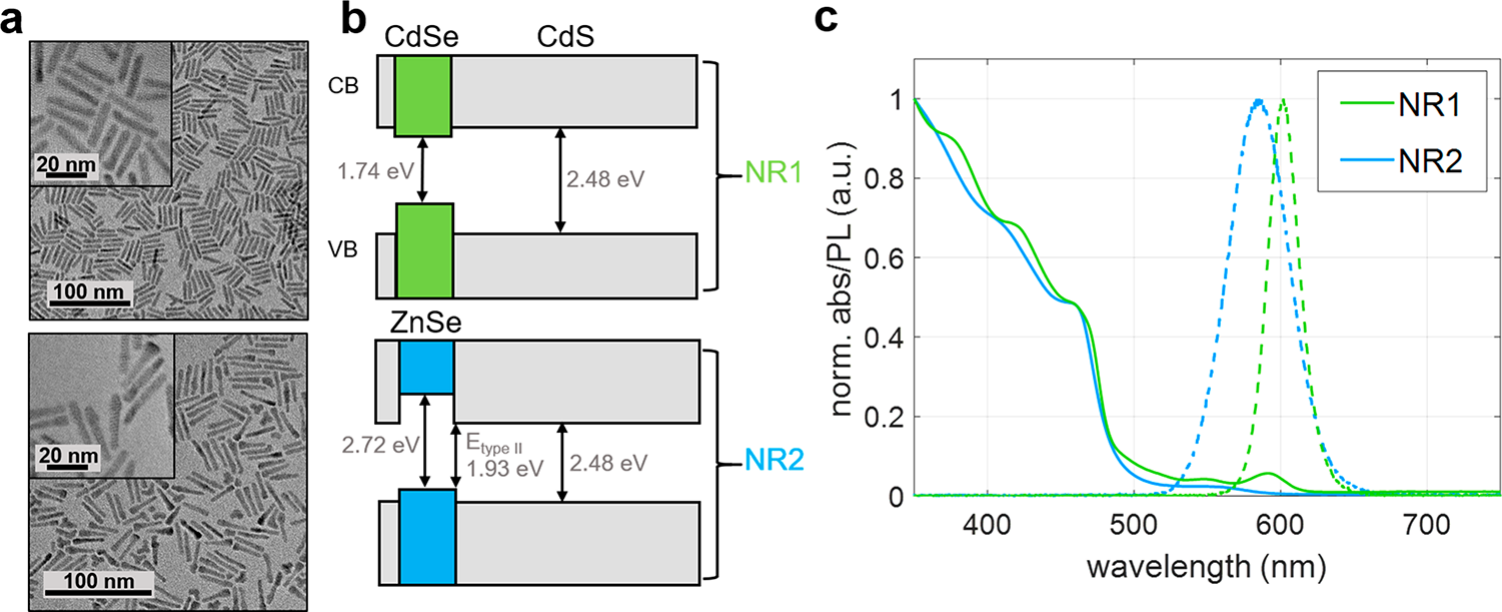
Synthesis characterization. (a) Transmission electron microscope (TEM) images for type-I^1^/_2_ CdSe/CdS seeded nanorods (NR1, top) and type-II ZnSe/CdS seeded nanorods (NR2, bottom). Insets are high resolution TEM images. Scale bars are 100 nm (20 nm in insets). (b) Energy diagrams of NR1 (top) and NR2 (bottom) showing the band gaps of all constituent materials and the type-II band-edge transition (bottom). (c) Normalized absorbance (abs, solid lines) and photoluminescence (PL, dashed lines) of NR1 (green) and NR2 (blue). Emission peaks are at ∼600 nm and ∼583 nm, respectively.

The experimental setup is demonstrated in Figure 2a. Briefly, a pulsed laser beam is focused on a single particle by a high numerical aperture objective lens. Excitation intensity is well below saturation, at ⟨*N*⟩ ≈ 0.1 (mean number of photons absorbed per pulse; see SI section S2). The NR fluorescence is collected via the same objective lens and imaged onto a 23-pixel SPAD array detector (SPAD23, Pi Imaging technology), shifted by ∼2 Rayleigh ranges from the image plane to create a defocused image. The shift magnitude and sign are crucial to create the defocused pattern, as discussed in ref (1) and in SI section S3. The detector array contains 23 pixels (each pixel is an independent single-photon detector) arranged in a hexagonal lattice (Figure 2b, top) and connected to time-to-digital converters (TDCs) implemented on a field-programmable gate array (FPGA). Each detected photon is time-stamped with a precision of ∼100 ps (full width at half-maximum) and address-stamped according to the pixel it was detected in. Intensity and temporal corrections are applied following refs (20) and (26), with adaptations to the SPAD23 detector, and detailed in SI section S4. Notably, the high temporal resolution allowed filtering out detection pairs originating in interpixel optical crosstalk by temporal gating rather than a statistical correction.

**Figure 2:**
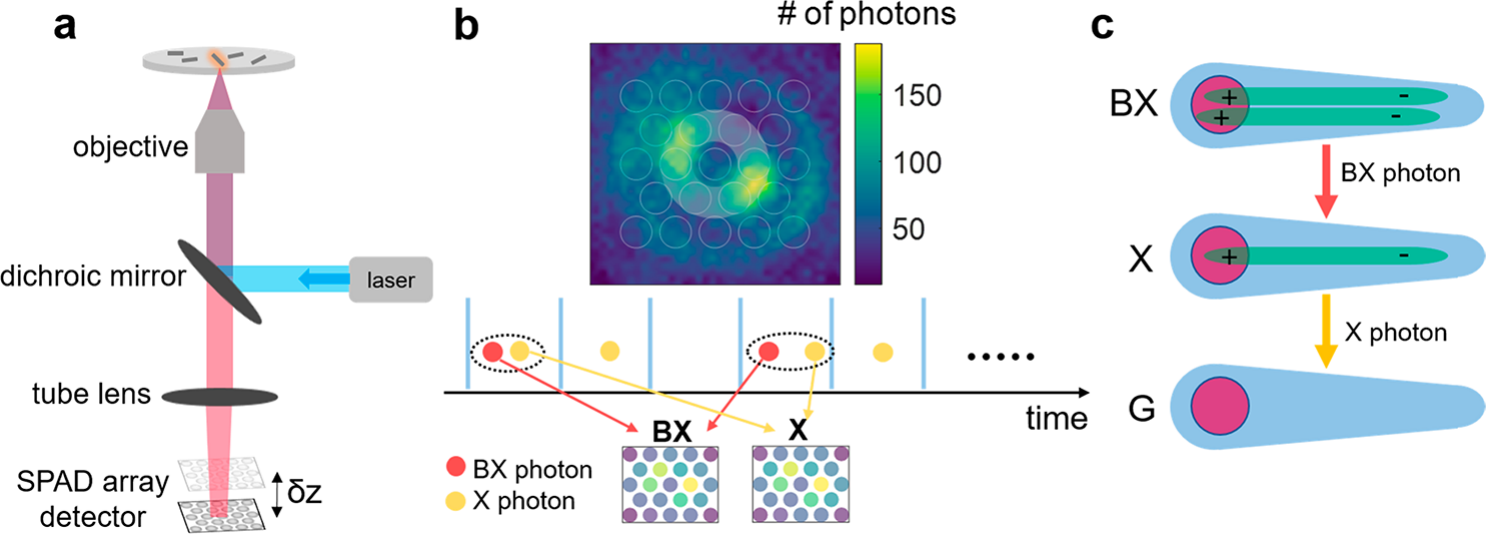
Optical setup and heralded defocused imaging technique. (a) Schematic of the defocused imaging setup consisting of an inverted microscope with a 470 nm laser excitation, a dichroic mirror, an objective lens, and a tube lens coupled to a single-photon avalanche detector array of 23 pixels (SPAD23). Each detected photon is identified with a pixel number and an arrival time. (b) Top: defocused image of a single NR imaged with a CMOS camera. The overlaid 23 white circles represent the collection area of the 23 pixels of the SPAD array. The white shaded area highlights the six-pixel ring used for anisotropy estimation. Bottom: Illustration of the heralded spectroscopy analysis that postselects photon pairs (indicated by black dotted ellipses) and sorts them into two groups: (i) first arriving photons (i.e., biexciton photons, filled red circles) and (ii) second arriving photons (i.e., exciton photons, filled orange circles). Summing each group of photons over all pixels yields emission maps of the biexciton and exciton states individually, as shown at the bottom. (c) A sketch of biexciton emission cascade of a doubly excited seeded nanorod. The cascade features two subsequent transitions, defining the first emitted photon as a biexciton photon (red colored arrow) and the second emitted photon as an exciton photon (orange colored arrow). BX, X, and G correspond to biexciton, exciton, and ground states.

The magnification was chosen such that the center ring of the defocused pattern falls on the six pixels around the detector center (highlighted in Figure 2b). This ring contains most of the information about the in-plane dipole orientation, and hence, these six pixels are used in the subsequent analysis. Heralded isolation of BX and X emission was done following the scheme described in refs (20) and (21). Briefly, the high temporal resolution of the detectors allows isolating emission cascades originating in cascaded relaxation from the BX to the X to the GS state (BX–X–GS, illustrated in Figure 2c) from the overwhelmingly stronger singly excited fluorescence background, by postselecting photon pairs detected following the same excitation pulse (Figure 2b, bottom panel). The first photon of the pair is associated with the BX-to-X transition, and the second with the X-to-GS transition. Additionally, pairs with an interdetection delay shorter than 4 ns or where the first photon arrived more than 2 ns after the excitation pulse are filtered, to minimize the number of artificial pairs induced by optical crosstalk and dark counts, respectively (see SI section S4).

Figure 3 presents the results of the analysis described above for a 5 min measurement of a single representative NR. Figure 3a depicts the fluorescence intensity as a function of time for 20 s of the measurement, summed over all detector pixels. The contribution of dark counts is statistically estimated from prior characterization of the detector and subtracted from the raw measured intensity. The intensity trace shows a typical single-particle behavior, with fluctuations between well-defined “on” and “off” states. Figure 3b visualizes the intensity measured in each of the 23 pixels of the array integrated over the whole measurement. Notably, the anisotropy of the defocused pattern seen in Figure 2b is also evident in Figure 3b, despite the lower spatial sampling resolution. To quantify this anisotropy, and hence the level of polarization, the anisotropy of the six-pixel ring around the central pixel is estimated by a fit to a sum of a squared sine and an isotropic background:
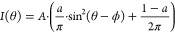
1*I*(θ) is the photon count rate (intensity) as a function of θ, the angle along the six-pixel circle, *A* is a normalization factor (the total number of photon pairs detected), *a* is the anisotropy, and ϕ is a global phase. The fit parameters are *a* and ϕ. The anisotropy value, *a*, is used as a measure of the emission anisotropy. While *a* is not a direct estimator of emission polarization, it is correlated with it and thus allows us to compare the dipole orientation and magnitude of the BX and X states, as described below. To consider the fact that the collection area of each pixel subtends roughly one-sixth of the circle, the values measured by the array pixels are fitted to the integral of eq 1 over ±30°, which describes the photon count rate of a pixel detector centered at θ^′^:
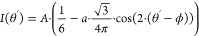
2The fit results for the specific NR featured in Figure 3e, considering all detected single photons, are *a*_*All*_ = 0.48 ± 0.04 and ϕ_*All*_ = 118° ± 3° (all errors in the paper are estimated as the 68% confidence interval of the fit). The angle ϕ extracted from the fit represents the in-plane orientation of the transition dipole, which is aligned along the NR, and conforms with the emission patterns appearing in Figure 3b–d.

**Figure 3:**
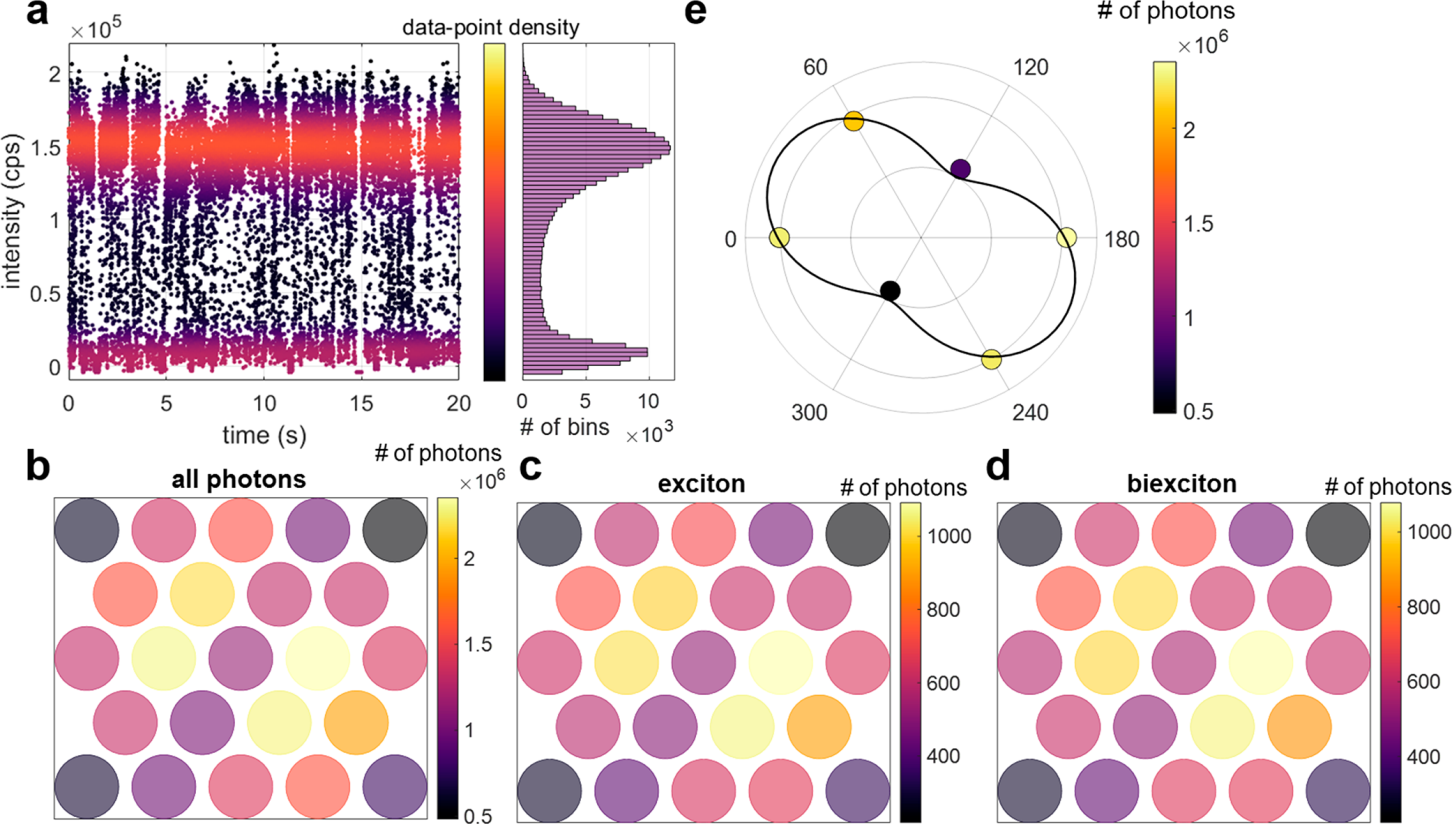
Heralded defocused imaging analysis of a single nanorod. (a) Left: total fluorescence intensity collected for all pixels as a function of measurement time in a 20 s time window. Each data-point represents the average intensity over a 1 ms time bin, colored according to the local density of data-points for clarity, and corrected for detector dark counts. Right: histogram of intensity values over a 5 min measurement revealing the “on” and “off” states, evident as peak occurrences at high and low intensity, respectively. (b–d) Histograms, by pixel, of all detected photons (b), and postselected exciton (c) and biexciton (d) detections from a 5 min measurement of a single type-II ZnSe/CdS seeded nanorod (NR2), applying heralded defocused imaging. Color scale represents the number of detections at a given detector pixel. (e) Polar representation of the intensity values detected by the six pixels of the inner ring of the array (highlighted in Figure 2b, top) for all detected photons in the measurement along with the fit (black solid line) to the integrated dipole emission model. The six data points are colored according to the number of detected photons in each pixel. The same fitting process is applied for the exciton and biexciton data sets and is presented in section S5 of the SI.

Applying the heralded isolation of BX–X–GS emission cascades, as described above (Figure 2b, bottom), to the same measurement yields ∼11,000 postselected BX–X pairs. The first photon of each pair can be associated with BX emission, and the second with X emission. Repeating the emission pattern anisotropy estimation for the postselected X-to-GS and BX-to-X yields the histograms shown in Figure 3c and 3d, respectively. The fit results for the BX and X are *a*_*BX*_ = 0.44 ± 0.05, ϕ_*BX*_ = 119° ± 3°, *a*_*X*_ = 0.45 ± 0.05, and ϕ_*X*_ = 118° ± 3°, respectively. Note that since the X emission dominates the overall emission, we expect to get similar anisotropy values for the X (*a*_*X*_) and for all of the photons in the measurement (*a*_*All*_). The pixel dead time leads to an apparent reduction in both X and BX anisotropy values due to pairs of photons that impinged on the same pixel. The reported X and BX anisotropy values in Figure 3 and 4 are statistically corrected for this small bias (see SI section S4). Importantly, this correction affects the X and BX anisotropy values equally and does not affect the difference between them. It is worth noting that the high-temporal resolution single-photon detection allows for many more postanalyses and insight from the same raw data. Other examples of single-particle analyses of NR1 and NR2 can be found in SI section S6.

**Figure 4:**
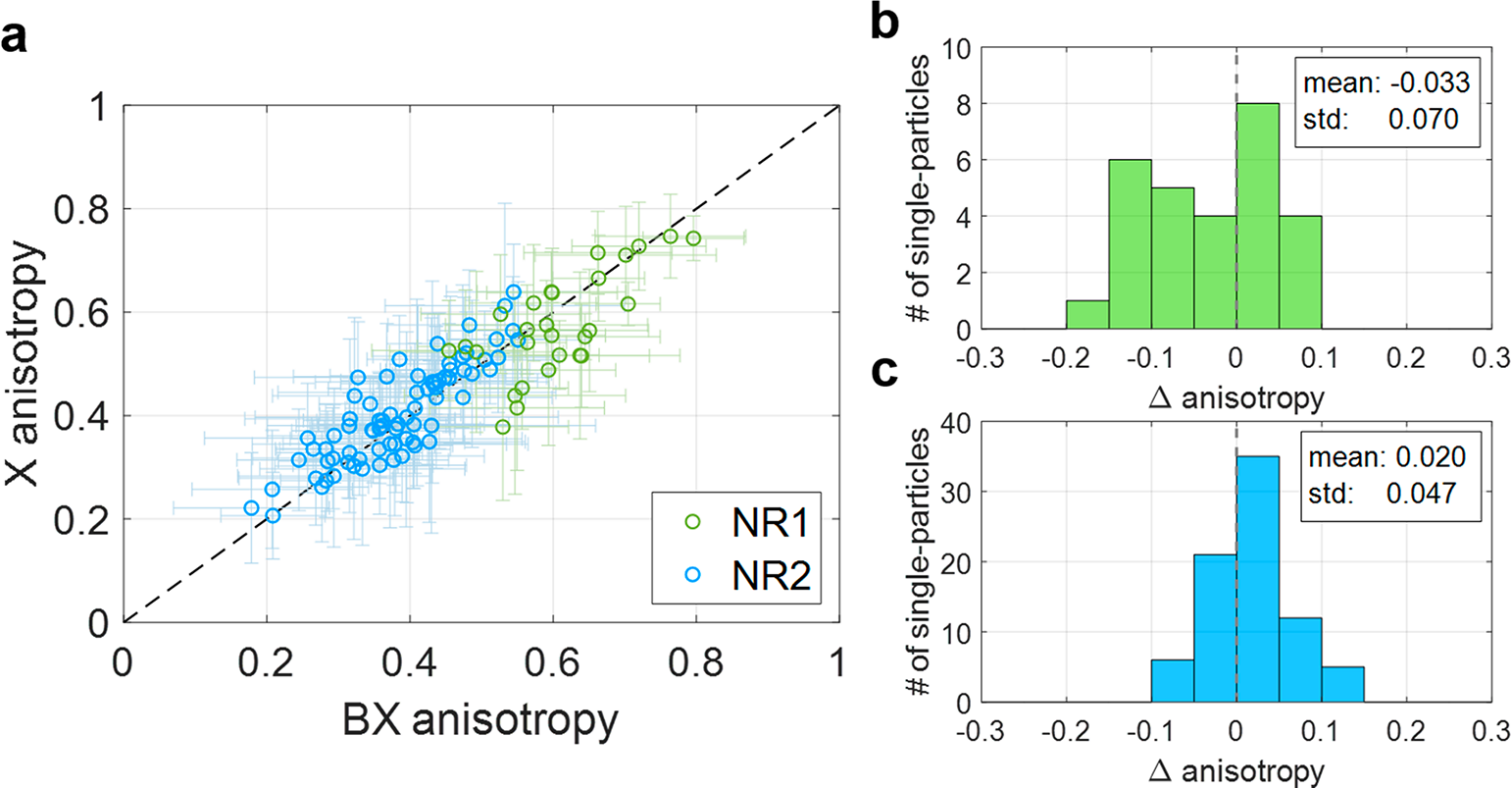
Aggregate X and BX emission anisotropy analysis. (a) Exciton emission anisotropy versus biexciton emission anisotropy, extracted by heralded defocused imaging, for all measured single NCs: 28 type-I^1^/_2_ CdSe/CdS (NR1, green rings) and 79 type-II ZnSe/CdS (NR2, blue rings) seeded nanorods. The dashed black line in all panels is a guide to the eye, indicating the same anisotropy values for both X and BX photons. (b–c) Histograms of Δ_*anisotropy*_ = *X*_*anisotropy*_ – *BX*_*anisotropy*_ for NR1 (b) and NR2 (c).

The heralded single-particle analysis described above was repeated for 28 NR1 and 79 NR2 nanocrystals. The aggregate results are presented in Figure 4a. It is evident that the anisotropy of the BX and X emission (*a*_*BX*_ and *a*_*X*_) is highly correlated. In fact, for each single NR measurement, the values extracted for the BX and X are within the fit confidence intervals of each other (close to the diagonal in Figure 4a). The orientations of the BX-to-X and X-to-GS transition dipoles (ϕ_*BX*_ and ϕ_*X*_) are also aligned, within the fit error, for all single NRs (see SI section S7). These similarities can be expected, given the twofold degeneracy of the lowest excited state in these NCs.^27^ However, exciton—exciton interaction can somewhat alter the properties of the BX emission, as is well-known for the BX emission spectrum.^20^ Indeed, further analysis of the results, clustered by NR type, reveals small but statistically significant deviations of the BX-to-X transition dipole anisotropy from that of the X-to-GS transition. Aggregate analysis of NR1 (Figure 4b) reveals that BX emission is typically more anisotropic than the X for NRs of this type. The statistical significance of this observation is confirmed via a paired Student’s *t* test, yielding a score of 2.5 (corresponding to a *p*-value of ∼0.02). NR2 shows the opposite trend, where BX emission is less anisotropic than X emission (Figure 4c), with a paired Student’s *t* test score of 3.8 (corresponding to a *p*-value of ∼0.0003).

The experimental results presented above indicate a small yet statistically significant deviation of the anisotropy of the BX-to-X emission from that of the X-to-GS emission. In the following, we discuss two mechanisms governing X-to-GS emission anisotropy: dielectric anisotropy and exciton fine structure. We propose how these mechanisms might lead to the observed results.

The dielectric contribution to emission anisotropy in elongated NCs was discussed extensively in past literature, relating the increased probability to emit light polarized parallel to the long axis of the NR to the different boundary conditions of the electric field parallel and perpendicular to the rod surface.^6,28−30^ This effect can be estimated by approximating the NRs as homogeneous dielectric ellipsoids using an effective medium calculation.^31−33^ However, the emission anisotropy of the BX state is different, as light emission occurs against an existing spectator exciton background. The absorption bleach, induced by the presence of the additional exciton, implies a change in the effective refractive index of the NR. Indeed, Tanghe and co-workers recently reported on a sizable phase modulation of light passing through a sample of CdSe nanoplatelets (NPLs) upon excitation of the NPLs, which is proportional to a refractive index change. In their work, TA measurements were translated into transient refractive index data, estimating the refractive index modulation shortly after excitation. Upon photon absorption, the imaginary part of the complex refractive index, the extinction coefficient, is transiently changed. The Kramers–Kronig relations, connecting the real and imaginary parts of analytic complex functions, imply that also the real part of the refractive index must therefore change.^34^ As a result, the dielectric response of the material is altered according to the relation  (*ñ* and *ε̃* are the complex refractive index and complex dielectric constant, respectively). Tanghe et al. considered this effect in several spectral bands with respect to the band gap of the NPLs and showed that photoexcitation increases the refractive index on the high energy side of the absorption bleach (i.e., above the band gap) and decreases it on the low energy side. Here, despite the difference in the NC structure and composition, their results may be used as a guideline to estimate the magnitude of this effect. In their reported results, the refractive index change (Δ*n*) at the level of 1 excitation per NPL (⟨*N*⟩ = 1) measured at 600 nm (almost 100 nm red-shifted from the absorption edge) is ∼0.01. In our particles, both NR1 and NR2, the emission wavelength of 600 nm is also red-shifted by ∼100 nm from the main absorption edge; hence, we expect a similar change of refractive index following photoexcitation. Considering the dielectric parameters of CdS, the relative change in the refractive index corresponds to  for both NR1 and NR2 (since both have a CdS rod shell and the refractive index, *n*_0_, of CdS at 600 nm is 2.34^6^). The dielectric contribution to the anisotropy scales with square of the ratio of the internal electric field strength between the major and minor axis of the rod (). Following the derivation of Vezzoli et al.^6^ (see also section S8 in the SI), the relative change in the dielectric effect on emission anisotropy, , can be estimated to be ∼1%. The BX emission anisotropy is expected to be reduced by this amount because spectator X decreases the CdS refractive index at 600 nm.

In addition to the dielectric effect, the exciton fine structure also influences emission polarization. The intrinsic difference between the crystal structures of CdSe and ZnSe may explain the qualitatively different results for the two NR types. As both CdSe and CdS have a wurtzite crystal structure with hexagonal symmetry, the *c*-axis of the CdSe core aligns with that of the CdS shell.^6,35,36^ This may induce a stronger linear polarization along the rod, as is evident in the higher anisotropy values for NR1 (Figure 4a). The exciton fine structure of CdSe was extensively studied in previous studies.^6,25,31,37−41^ The eightfold degenerate exciton ground state of a spherical NC is greatly influenced by the morphology, crystal structure anisotropy, and electron–hole exchange interaction. All mentioned factors may alter the splitting, ordering, and transition oscillator strengths of the states.^38^ The shape anisotropy, together with the exchange interaction, split the eightfold degenerate exciton into five levels, three of which are twofold degenerate. For elongated CdSe NRs, exchange interactions make 0^*u*^ the lowest optically active exciton state.^6^ The 0^*u*^ state features the observed 1D dipole emission, aligned with the elongated dimension of the NR. It can be assumed that, for NR1, the overlap between the hole and electron wave functions of the BX is stronger than that of the X due to the stronger Coulomb attraction to the two holes confined in the CdSe core. The higher overlap may increase the exchange interaction and hence the splitting of the fine structure. Enhanced splitting means emission will be even more dominated by the lowest fine structure state, thus increasing the BX anisotropy. The effect of the fine structure on the BX emission anisotropy is much smaller in the case of NR2 due to the charge separation of the excited electron and hole, which dramatically reduces the electron–hole exchange interaction. To summarize this comparison, the dielectric effect is expected to reduce the anisotropy of the BX emission (since emission is red-shifted from the absorption edge), whereas the increased electron–hole overlap of the BX is expected to increase the BX emission anisotropy. The former has a similar influence on both type-I^1^/_2_ and type-II NRs, while the latter predominantly affects the type-I^1^/_2_ NRs. This might explain why the type-I^1^/_2_ NR1 shows increased BX emission anisotropy compared to the X (fine-structure dominated), while the type-II NR2 has decreased BX anisotropy (dielectric-effect dominated). Interestingly, we have also studied a sample of shorter type-II NRs (see SI section S9), which exhibited a similar trend as the longer type-II NRs (NR2) showing a slightly lower emission anisotropy for the BX compared with the X. Due to lack of statistics, no significant conclusion can be deduced for these shorter NRs.

It is worth noting that other methods can also probe the photon polarization correlation in emission cascades. A seminal example is a work by Aspect et al., demonstrating a violation of Bell’s inequalities in the polarization correlation of photon pairs emitted in a radiative cascade of calcium.^42^ The system applied there consists of a beamsplitter splitting light into two paths; each path is further divided between two single-photon detectors by rotatable polarizing beamsplitters, allowing estimation and correlation of the polarization in the two arms. While extracting quantitative polarization values is more challenging with heralded defocused imaging, as performed here, it introduces several significant advantages over the above-mentioned technique. Heralded defocused imaging obviates the need to repeat the experiment at multiple polarizer orientations, as it is sensitive to all polarization orientations at once. The detection and timing setup, consisting of a single compact component (the SPAD array detector) placed at the defocused image plane, is significantly simpler than those of traditional multiplexed polarimeters. Finally, the most critical advantage is the potential for straightforward adaptation to higher-pixel-count SPAD arrays. This up-scaling will allow extracting the dimensionality and 3D orientation of multiexcitonic transition dipoles, as previously demonstrated with imaging detectors for the singly excited state.^1,16,43^

We present a new approach to directly probe the transition dipole moment of the BX-to-X transition in single NCs. Heralded defocused imaging provides us with the dipole orientation mapping of single NRs onto a 2D SPAD array detector to temporally differentiate between the emission transition dipole moments of the first and second excited states. The results reveal variations between the emission transition dipole moment of the two first excitonic states, showing higher BX emission anisotropy for the type-I^1^/_2_ NRs and lower BX emission anisotropy for the type-II NRs compared with the X emission anisotropy. We discuss possible transient deviations of refractive index and exciton fine structure present in the BX state and how they may explain these observations. The heralded defocused imaging technique introduced here expands the limited set of experimental tools that directly probe multiply excited states in single semiconductor nanocrystals. It can be easily realized and applied to various nanocrystal systems, and scaling up the number of detector pixels can further support 3D polarization analysis. Heralded defocused imaging demonstrates the benefit of harnessing photon correlations to investigate multiply excited states in semiconductor nanocrystals by uncovering previously inaccessible insights and holds great potential to further our understanding of these materials.

## Supporting Information Available

Details of the nanorods including synthesis, characterization methods, sample preparation, and saturation estimation assay; details of the defocused single-particle spectroscopy setup; dark count rate correction, crosstalk estimation, and detector dead-time correction details; complementary anisotropy analysis for the exciton and biexciton; additional examples of single-particle measurements; exciton-biexciton in-plane angle correlation details; dielectric effect estimation details; details of an additional measured sample. (PDF)
