# Supplementary material for: Resolving the Emission Transition Dipole Moments of Single Doubly Excited Seeded Nanorods via Heralded Defocused Imaging
Source: Nano Lett. 2023 Jun 8;23(12):5417–23. doi: 10.1021/acs.nanolett.3c00155 (PMC10311519; doi:10.1021/acs.nanolett.3c00155)
Supplement: Supplementary file 1 — nl3c00155_si_001.pdf [file nl3c00155_si_001.pdf]

Supporting Information: Resolving the emission transition dipole moments of single doubly-excited seeded nanorods *via* heralded defocused imaging

Daniel Amgar[1,†], Gur Lubin[1,†], Gaoling Yang[2], Freddy T. Rabouw[3], and Dan Oron[4,]*

[1] Department of Physics of Complex Systems, Weizmann Institute of Science, Rehovot 76100, Israel

[2] School of Optics and Photonics, Beijing Institute of Technology, Beijing 100081, China

[3] Debye Institute for Nanomaterials Science, Utrecht University, Princetonplein 1, 3584 CC Utrecht, The Netherlands

[4] Department of Molecular Chemistry and Materials Science, Weizmann Institute of Science, Rehovot 76100, Israel

S1: Materials and methods

Details of the dot-in-rods synthesis

Chemicals

Hexadecylamine (HDA, 98%, Aldrich), diethylzinc (Et_2Zn, 1 M solution in hexane, Aldrich) Cadmium oxide (99.99%, Aldrich), Sulfur (99.999%, Aldrich), Selenium (99.999%, Aldrich), oleic acid (OA, 90%, Aldrich), trioctylphosphine (TOP, 90%, Aldrich), dodecylamine (98%, Fluka), trioctylphosphine oxide (TOPO, technical grade, 99% Aldrich), 1-octadecene (ODE, technical grade, 90% Aldrich), hexylphosphonic acid (HPA, 99%, PCI), n-octadecylphosphonic acid (ODPA, 99%, PCI), methanol (anhydrous, 99.8%, Aldrich), hexane (anhydrous, 99.9%, Aldrich), toluene (99.8%, Aldrich). All chemicals were used as received without any further purification.

Synthesis of CdSe NCs

TOPO (3.0g), ODPA (0.280g) and CdO (0.060g) are mixed in a 50mL flask, heated to 150°C and exposed to vacuum for 1.5 hour. Then, under nitrogen, the solution is heated to above 370°C to dissolve the CdO until it turns optically clear and colorless. At this point, 1.8 mL of TOP is injected in the flask and the temperature is allowed to recover back to 370°C. At this temperature, Se:TOP solution (0.058g Se + 0.5 mL TOP) was injected and the heating mantle is removed to stop the reaction after 2 minutes. After the synthesis, the nanocrystals are precipitated with methanol, they are washed by repeated redissolution in toluene and precipitation with the addition of methanol, and they are finally dissolved in toluene.

Synthesis of CdSe/CdS dot-in-rod

This synthesis was adapted from the previously reported procedure in the literature.[3] Firstly, 0.09 g CdO, 3 g TOPO, 0.08 g HPA and 0.290 g ODPA were dissolved and vacuumed in advance at 150°C. The resulting solution was allowed to be heated to 350 °C under nitrogen and 1.5 g TOP was injected. Then the mixture was heated to 380 °C and propriate amount of CdSe seed together with 0.12 g S in 1.8 mL TOP were injected simultaneously. The length of CdS was controlled by adjusting the concentration of CdSe seed. The mixed solution was maintained at ~380 °C for 8 min under nitrogen to complete the growth of the CdSe/CdS dot-in-rods, and the heating mantle was moved to stop the reaction. The CdSe/CdS dot-in-rods were further purified with toluene and methanol as solvent and nonsolvent, then dispersed and stored in toluene.

Figure S1a shows size distributions of the length and width of ~200 CdSe/CdS NRs (NR1).

Synthesis of ZnSe NCs

9.4 g of HDA was degassed under vacuum at 120 °C in a reaction flask, under argon flow, the mixture was heated up to 310 °C. Then, a mixture of 1 mL 1.0 M selenium dissolved in TOP, 0.8 mL diethylzinc and 4 mL TOP, was quickly injected. The reaction was continued at a constant temperature of 270 °C for 25 min and then cooled to room temperature.

Preparation of Cadmium and Sulfur Stock Solutions

0.034 M cadmium oleate was prepared by mixing 0.03 g (0.24 mmol) CdO in 0.6 mL oleic acid and 6.4 mL ODE. The solution was heated to 280 °C under argon flow with rigorous stirring until all of the CdO dissolved. 0.29 M S solution was prepared by adding 23.3 mg sulfur in 2.5 mL of dodecylamine at ~40 °C.

CdS Shell Synthesis

ZnSe/CdS core-shell nanocrystals (NCs) were synthesized following a previously published procedure with some modifications.[1] For typical CdS shell coating, 1.1 g unprocessed ZnSe cores, 5.3 mL octadecene (ODE) were loaded into a 50 mL three-neck flask. The solution was degassed at 100 °C. After that the solution was heated to 240 °C under argon, a mixture of 0.6 mL of 0.034 mmol/mL cadmium oleate stock solution and 0.06 mL of 0.29 mmol/mL sulfur stock solution was injected continuously at 0.72 mL/h. After the injection was finished, the mixture was further annealed for 5 min at 240 °C and then cooled down to room temperature.

ZnSe/CdS-CdS NRs Synthesis

This synthesis was adapted from the previously reported procedure in the literature.[2,3] In a typical synthesis CdO (60 mg), ODPA (290 mg) and HPA (80 mg) are mixed in TOPO (3.0 g). The mixture is degassed under vacuum at 150 °C for 90 min. After degassing step, the solution was heated to 380 °C under argon until it became clear, then 1.8 mL of TOP was injected and the temperature was recovered to 380 °C. Subsequently a solution of 120 mg S in 1.8 mL TOP mix with 40 nmol ZnSe/CdS nanocrystals is rapidly injected. Then the growth was stopped after nanorods grow for 8 min at 365 °C. The NRs were precipitated with methanol and dispersed in toluene.

Figure S1b shows size distributions of the length and width of ~200 ZnSe/CdS NRs (NR2).

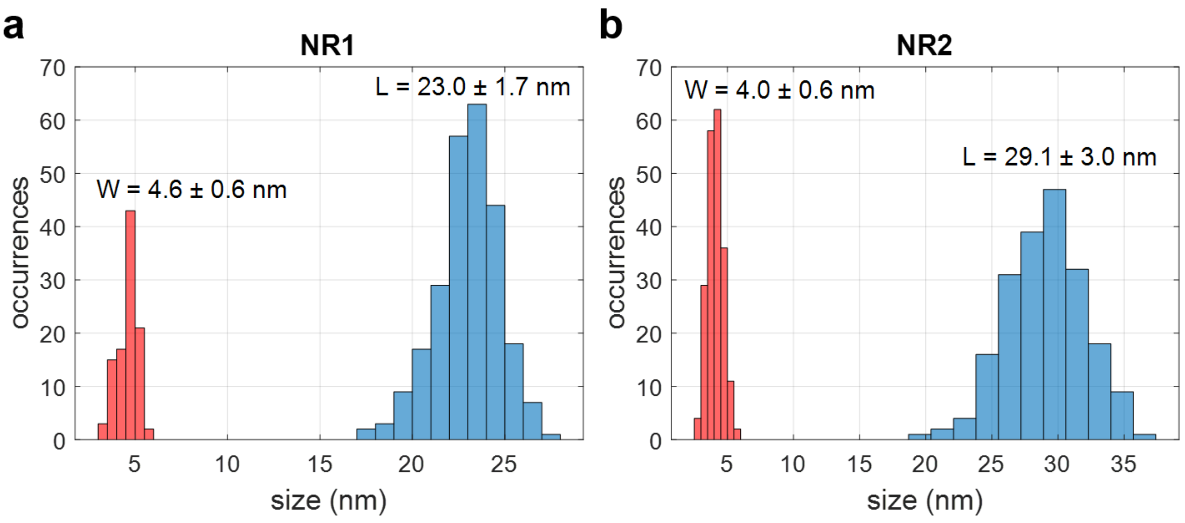

Figure S1. Size distributions of the nanorods. Histograms of the distribution of length (L, blue bars) and width (W, red bars) of CdSe/CdS dot-in-rods (NR1) and ZnSe/CdS dot-in-rods (NR2). These length and width size distributions were extracted from transmission electron microscope (TEM) images for ~200 NRs, and estimated using imageJ software.

Characterization methods

TEM images were taken on a JEOL 2100 TEM equipped with a LaB6 filament at an acceleration voltage of 200 kV on a Gatan US1000 CCD camera. UV-vis absorption spectra were measured using a UV-vis-NIR spectrometer (V- 670, JASCO). The fluorescence spectrum was measured using USB4000 Ocean Optics spectrometer excited by a fiber coupled 407 nm LED in an orthogonal collection setup.

Sample preparation

The samples for the single-particle experiments were prepared as follows. A stock solution of DiR nanocrystals was diluted in a solution of poly(methyl methacrylate) (PMMA, Aldrich) in Toluene by $10^4 - 10^6$. The PMMA/Toluene solution was prepared by dissolving 3 wt% of PMMA powder in Toluene. The solution was gently stirred and heated to ~65° overnight. To prepare the sample for the microscope, 200 µl of the PMMA/Toluene

solution were spin coated onto a glass coverslip in two steps; (i) 5 seconds at 800 rpm, (ii) 45 seconds at 2000 rpm.

S2: Saturation experiments

To avoid possible contamination of the results with emission from higher-multiexcitonic states (triexcitons and above), illumination power was kept far below saturation. At these illumination intensities the probability of absorbing 3 photons or more in a single pulse and a radiative decay of the triexciton or higher states is negligible. The saturation power, for which $\langle N \rangle$, the average number of photons absorbed per pulse, equals unity, was estimated following the scheme described in the supporting information of ref 4: A single particle was illuminated by the same setup described in the main text. During the measurement, the laser power was increased every 10 s, in 10 equally-spaced power steps up to some maximal power and then lowered back down to the starting point. In the measurement featured in figure S2, the power range was from $\sim 20\ nW$ to $\sim 700\ nW$. Figure S2a presents the intensity trace for a representative measurement showing the ascending and descending intensity as a function of measurement time with the power steps, as well as the 'on'-'off' blinking mentioned in the main text (in relation to figure 3a). To construct the saturation curve, we considered both time windows with the same power (from the ascent and descent) and extracted the 'on' state typical intensity for that illumination power (the peak occurrence intensity, seen as the brighter, higher-density regions in figure S2a). This isolation of the 'on' state peak is aimed to avoid bias associated with possibly enhanced blinking at higher intensity excitations, as described in the supporting information of ref 4. The saturation curve is shown in figure S2b with a fit (solid red line) to a saturation function:

$$P = a \cdot \left(1 - e^{-\frac{I}{Isat}}\right), \tag{S1}$$

Where P is the 'on' state peak, a is asymptotic 'on' state peak, I is excitation power, and I_{sat} is the saturation power. The data in figure S2b agree well with the suggested model and the saturation power extracted from the fit is $I_{sat} = 670 \pm 70\, nW$ (68% confidence interval, green dashed line). The excitation power used in this work is $I_{exc} = \sim 80\, nW$ (purple dashed line in figure S2b), which is far below the saturation power.

The probability to create n excitons per excitation pulse can be estimated from the Poissonian distribution:

$$(n) = \frac{\lambda^n \cdot e^{-\lambda}}{n!} \tag{S2}$$

$$\lambda = \frac{I}{I_{sat}} \tag{S3}$$

The probability that the NC will absorb at least one photon in an excitation pulse, at the excitation power used in this work, is thus ~11%. The probability to absorb at least two photons per pulse (creating a biexciton) is 0.66%, and of absorbing three or more photons is 0.026%.

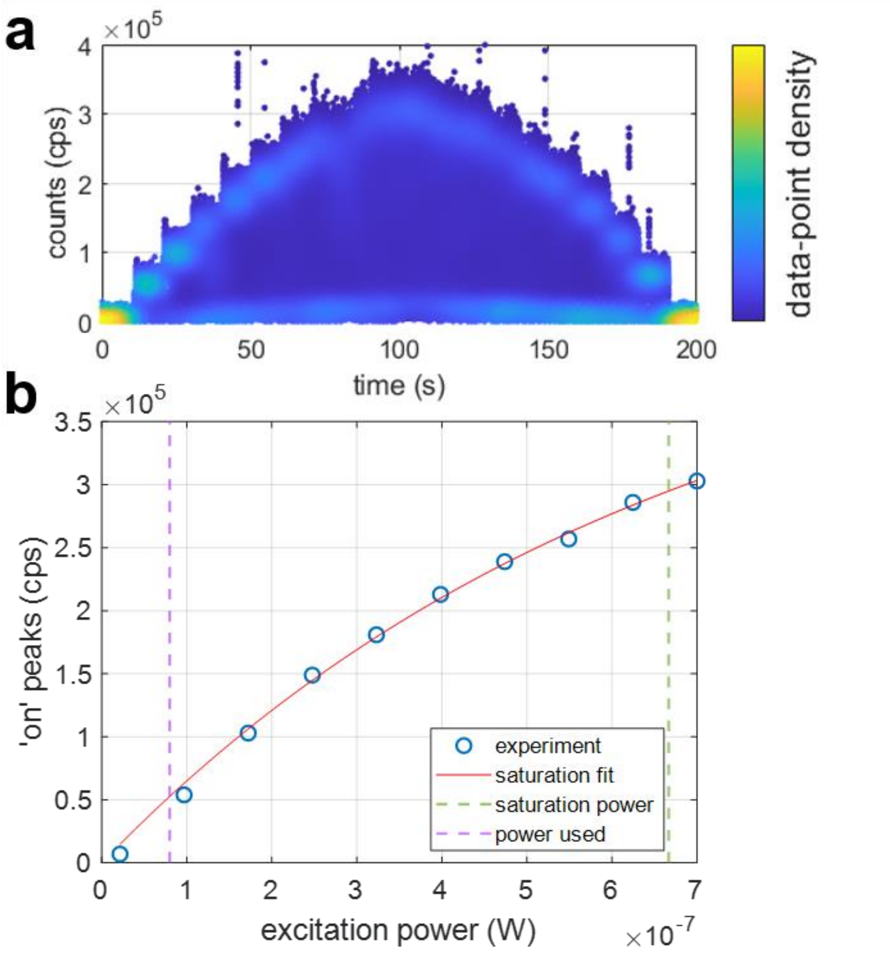

Figure S2. Saturation assay. (a) Intensity trace as a function of measurement time with 10 equally-spaced power steps ranging from 20 nW to 700 nW, where each step lasts 10 seconds. The power is gradually increased up to the maximal power and decreased back to the starting power. (b) Saturation curve presenting the "on" peak intensity as a function of excitation power fitted to a saturation function (red solid line). The saturation power and the power used in the experiments are marked by green and purple dashed lines, respectively.

S3: Defocused single-particle spectroscopy setup

The experimental setup (figure 2a in the main text) is built around a commercial inverted microscope (Eclipse Ti-U, Nikon). A 70 ps pulsed diode laser (LDH-P-C-470B, PicoQuant) provides a collimated beam at a wavelength of 470 nm and a repetition rate of 5 MHz. The linearly polarized laser is converted to circular polarization (using a $\lambda/4$ waveplate) to obtain uniform excitation of NRs, irrespective of orientation. The beam is

focused by a high numerical aperture (NA) oil immersion objective lens (x100, 1.3 NA, Nikon), which also collects the resulting fluorescence light. An additional magnification step inside the microscope results in magnification of x150. Back-scattered laser light is filtered out by a dichroic mirror (FF484-FDi02-t3, Semrock) and a long pass dielectric filter (BLP01-473R, Semrock). We note that the collected fluorescence is transmitted through the dichroic mirror so no effect of the dichroic on polarization is expected. Moreover, defocused imaging measurements image the k-space and are not sensitive to any polarization modification that may be introduced by the optical system. The defocused imaging path contains a 75 mm focal length relay lens that de-magnifies the image by a factor of two, for an overall magnification of ×75. An equivalent imaging path leading to a complementary metal-oxide-semiconductor (CMOS) camera (BFS, FLIR) is available using a flip mirror, allowing wide field imaging used for imaging the sample and aligning a single NC with the excitation beam before each measurement. Finally, the desired defocused emission pattern is imaged onto a 23-pixel single-photon avalanche diode (SPAD) array, fabricated in CMOS technology (SPAD23, Pi Imaging Technology). The detector pixels are organized in a hexagonal lattice with a pixel pitch of 23 μm (nearest neighbors). At the detector plane, the estimated diffraction-limited spot diameter is ~20 μm and the defocused pattern diameter is ~ 220 μm. The SPAD is attached to an XYZ translation stage to modify the defocus distance and for alignment. A field-programmable gate array (FPGA) with a coarse clock system, and an implemented array of time-to-digital converters (TDCs) with a fine resolution of ~10 ps (both synchronized with the laser excitation), assigns timestamps and pixel addresses to single-photon detections in the 23

pixels of the array. The trace of detections is then analyzed by a dedicated MATLAB script, implementing temporal and intensity corrections and analysis schemes.

S4: Corrections

As described previously in refs 4–6, two sources of artificial photon detection pairs must be considered when analyzing photon correlations with SPAD arrays. The first is pairs where at least one detection originates from the detector's dark counts rather than a fluorescence photon. The second is where the false photon pair detections arise from inter-pixel crosstalk. As described below, both sources of artificial pairs feature very different temporal responses from ‘true’ BX-X photon pairs and hence can be filtered by applying temporal gating. Residual dark count-induced artificial pairs are corrected statistically. Additionally, ‘true’ BX-X photon pairs that impinged on the same detector pixel are not registered by the system due to pixel dead time. The number of these undetected pairs is estimated from the measurements and added to the results. These three intensity corrections are discussed below. The final part of this section describes the effect of the mentioned corrections on the analyzed results.

Dark counts

The SPAD array detector features some probability for false photon detections, even when no fluorescence photons impinge on the sensor. These detections are known as dark counts. In this work, the dark count rate (DCR) is negligible compared to the single-photon detection rate (except for one 'hot' pixel) but comparable to the estimated rate of detected BX-X photon pairs.

Unlike the signal probability that decays exponentially with the delay from each excitation pulse, DCR is time-independent. Hence, the time-gating described in the main text filters out a significant portion of dark counts, while retaining almost all of the fluorescence data, leading to a better signal-to-noise ratio (SNR). Specifically, only photons arriving within the first ~2 ns following any excitation pulse are considered for labelling as BX emission (due to the fast decay of the doubly-excited state). As the laser period is 200 ns, and dark counts are time-independent, this filters out ~99% of the potential dark count-induced detection pairs, where the BX is a dark count. Similarly, considering only detections within ~100 ns following a BX detection to label as X emission filters out ~50% of the dark count-induced detection pairs, where the X is a dark count.

The residual dark count-induced pairs left after temporal gating are corrected by the following scheme. Each pixel's DCR is pre-characterized by performing a 100 s intensity measurement in the dark (figure S3). For each single-NR measurement presented in the main text, the temporally-resolved intensity in each pixel is extracted by a standard time-correlated single-photon counting (TCSPC) analysis. Next, the number of false photon pairs consisting of at least one dark count is estimated for each measurement. This estimation is done by multiplying the probabilities of ‘true’ photon detections and dark count events to occur, for each pixel pair, within the temporal gates employed for the heralded post-selection described above. For the measurement featured in figure 3 of the main text, we estimate that out of the total 14535 detected pairs, the BX detection was a dark count in ~658, the X detection was a dark count in ~239, and both X and BX were

dark counts in ~2. Hence, the temporal gates helped mitigate the dark-count-induced pairs to under an order of magnitude below the BX-X pairs signal. Results shown in the main text are after subtracting these estimated values of dark-count-induced detection pairs from the raw measurement data of each pixel pair. The significance of minimizing the impact of dark counts (by temporal gating) before this statistical correction, is to avoid the additional shot noise associated with the higher photon counts. Since shot noise is isotropic, it can skew our estimation of anisotropy values for the X and BX.

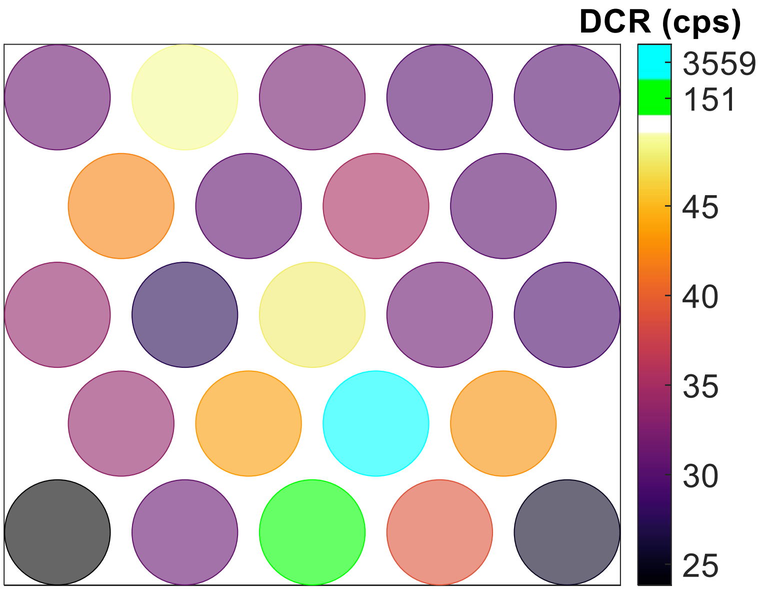

Figure S3. Dark count rate (DCR). The dark count rate of the detector pixels in counts per second (cps). The median DCR is ~32 cps. Note the ‘hot’ pixel at the second-to-bottom row with two orders of magnitude higher DCR than the median.

Crosstalk

The densely packed pixels give rise to inter-pixel optical crosstalk, attributed to photons emitted due to the detection process in one pixel and then detected by a neighboring pixel.[7] The SPAD array design mitigates crosstalk significantly[8], but the small residual crosstalk

probabilities are typically overwhelmingly higher than the probabilities for ‘true’ BX–X detections.[5] However, crosstalk and BX–X pairs feature separate time scales of inter-detection delay. Typical delays between a ‘true’ detection and the resulting crosstalk detection correspond roughly to the temporal precision of the detector (~100 ps FWHM), which is considerably shorter than the typical delay between BX and X emission, corresponding to the X fluorescence lifetime (τ >10 ns). Hence the requirement for at least 4 ns inter-detection delay applied in the heralded post-selection (see main text) filters almost all crosstalk detection pairs and just a small fraction of the BX–X pair signal.

The number of crosstalk-induced pairs in each measurement was estimated following the procedure detailed in ref 5, with some modifications. Briefly, to characterize crosstalk probabilities, the detector array is illuminated by a thermal light source (a halogen lamp), and the second-order correlation of photon arrival times, $G^{(2)}$, is extracted. The $G^{(2)}$ curve is expected to be flat for such a classical light source; however, a sharp peak is evident at short time-delays attributed to inter-pixel crosstalk. The excess photon pairs detected at short time delays are extracted for each pixel pair. These values are then divided by the number of overall single-photon detections in the pixel of the first detection to derive the time-resolved crosstalk probability for each pixel pair. Time-resolved crosstalk probability is the probability that, given a detection in one pixel, another pixel will register a crosstalk event at a given time delay. These probabilities are shown in figure S4a, where the rapid decay with time is evident. Figure S4b illustrates the effect of temporal gating in mitigating crosstalk contribution by plotting the crosstalk probability as a function of the inter-detection temporal gate (the minimal BX–X delay considered for the heralded analysis).

For the gate used in this work (4 ns, dashed line in figure S4b), the overall crosstalk probability is $\sim 2.28 \cdot 10^{-4}$ per detected photon, almost two orders of magnitude less than without gating. For the measurement shown in figure 3 of the main text, this translates to ~380 crosstalk pairs mistakenly labelled as BX–X cascades by the heralded post-selection ($\sim 1.66 \cdot 10^{6}$ single photons were detected within the 2 ns following each excitation pulse; $1.66 \cdot 10^{6} \cdot 2.28 \cdot 10^{-4} \approx 380$). We consider this value negligible compared to the overall 14535 post-selected BX-X cascades.

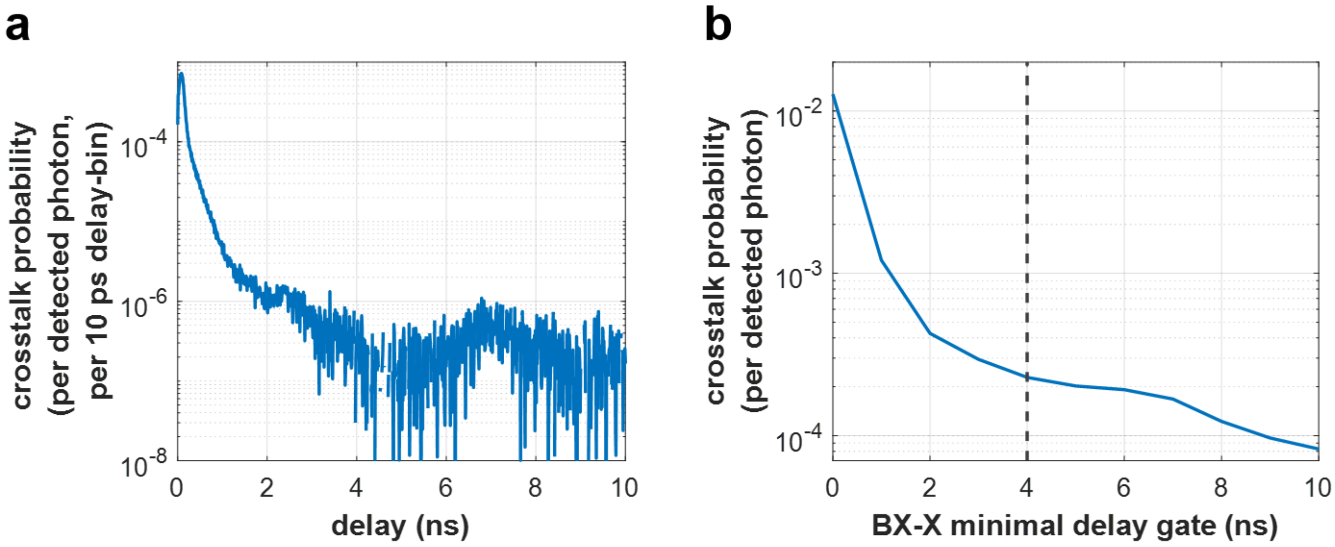

Figure S4. Crosstalk characterization. (a) Temporally resolved crosstalk probability. The vertical axis represents the probability for a crosstalk detection following any detection by the array, at the delay indicated on the horizontal axis. Delay-bins are 10 ps. (b) The crosstalk probability after applying the temporal gating as part of the heralded post selection. The vertical axis represents the probability for a crosstalk event to occur following a detection, at a delay longer than that indicated by the horizontal axis. This is generated by integrating the values in panel (a) from the BX-X minimal delay gate to infinity. Dashed line represents 4 ns, the temporal-gate used in this work, corresponding to a crosstalk probability of $\sim 2.28 \cdot 10^{-4}$, about two orders of magnitude below the crosstalk probability without gating ($\sim 1.27 \cdot 10^{-2}$).

Note that the critical difference from the crosstalk analysis in ref 5 is the detector's higher temporal precision, which allows resolving the order of detections in the crosstalk characterization and their inter-detection delay. This resolution is what allows the temporal

gating described above instead of a statistical correction. It also alleviates the need to assume symmetric crosstalk probabilities, as done in ref 5.

Same pixel pairs

If the BX and the X photons impinge on the same detector pixel, the second photon will not be detected (and hence the pair will not be identified). That is due to the pixel dead time, rendering each pixel non-active for ~50 ns following each detection. The number of BX–X photon pairs missed in this manner can be estimated from the collected data by the following method. Given a BX–X pair detection, the probability the X photon will be detected in pixel i, $pX(i)_0$, is calculated by dividing the number of X detections in pixel *i* by the overall BX–X pair number. $pBX(i)_0$, the probability to detect a BX photon in pixel *i* given a pair detection, is calculated similarly. These probabilities are slightly biased due to the dead time described above, affecting pixels where higher intensity was measured more. We can estimate the probability for the BX and the X photons to impinge on the same pixel, i, as $pX(i)_0 \cdot pBX(i)_0$, and thus correct the probabilities in the following way:

$$pX(i)_1 = pX(i)_0 + pX(i)_0 \cdot pBX(i)_0 \tag{S4}$$

$$pBX(i)_1 = pBX(i)_0 + pX(i)_0 \cdot pBX(i)_0 \tag{S5}$$

The results shown in the manuscript are all after applying this correction.

Visualizing the effect of the applied corrections

This section aims to describe the post-processing procedure done to the raw data measured, and demonstrates the effect of the data corrections discussed above step-by-step.

Figure S5 demonstrates the correction steps from the top panel to the bottom one for a single NR of the NR2 type (the same NR shown in figure 3 of the main text). In each panel, the intensity distribution along with the polar plots fitting the 6-pixel ring to the dipole function are presented, for “all single-photon detections”, BX and X (see the main text for more details). The top panel shows the raw data, without any correction. The second panel displays the same data after applying temporal gating on the BX (post-selecting only detections within 2 ns following the excitation pulse) and X (post selecting detections that are at least 4 ns and no more than 100 ns after the BX detection). The third panel shows the data after applying temporal gating and DCR correction, and the fourth panel presents the fully corrected results, including all three corrections: temporal gating, DCR, and dead-time compensation. Note that the result for all detected photons (“all photons”) is sensitive only to DCR correction. Temporal gating and dead-time corrections are part of the heralded analysis, and thus affect only the X and BX.

Two prominent observations in the top panel of Figure S5 (raw data) are the ‘hot’ pixel (the pixel with the highest counts that significantly deviates from the dipole model) and the lack of anisotropy for the X data set. The observation of relatively isotropic X is the result of crosstalk pairs, where a single detection is followed by crosstalk to a neighboring pixel, wrongly identified as BX–X pairs. The real detection (assigned to the BX) features anisotropy similar to “all photons”, while the crosstalk detection (assigned to the X) is expected to be more isotropic. The second panel (temporal gating correction) shows the

effect of filtering out the crosstalk pairs on the X anisotropy estimation. Additionally, the short temporal gating of the BX filters most of the BX dark counts, especially evident in the ‘hot’ pixel. The third panel (adding DCR correction to compensate for the residual dark counts) shows results with much better agreement to the dipole model, especially evident for the ‘hot’ pixel. The bottom panel (adding compensation for detection pairs missed due to pixel dead time) represents a relatively small correction leading to a slight increase in the anisotropy estimation of the BX and X. This bias reflects the larger number of missed photon pairs estimated at pixels with higher photon counts.

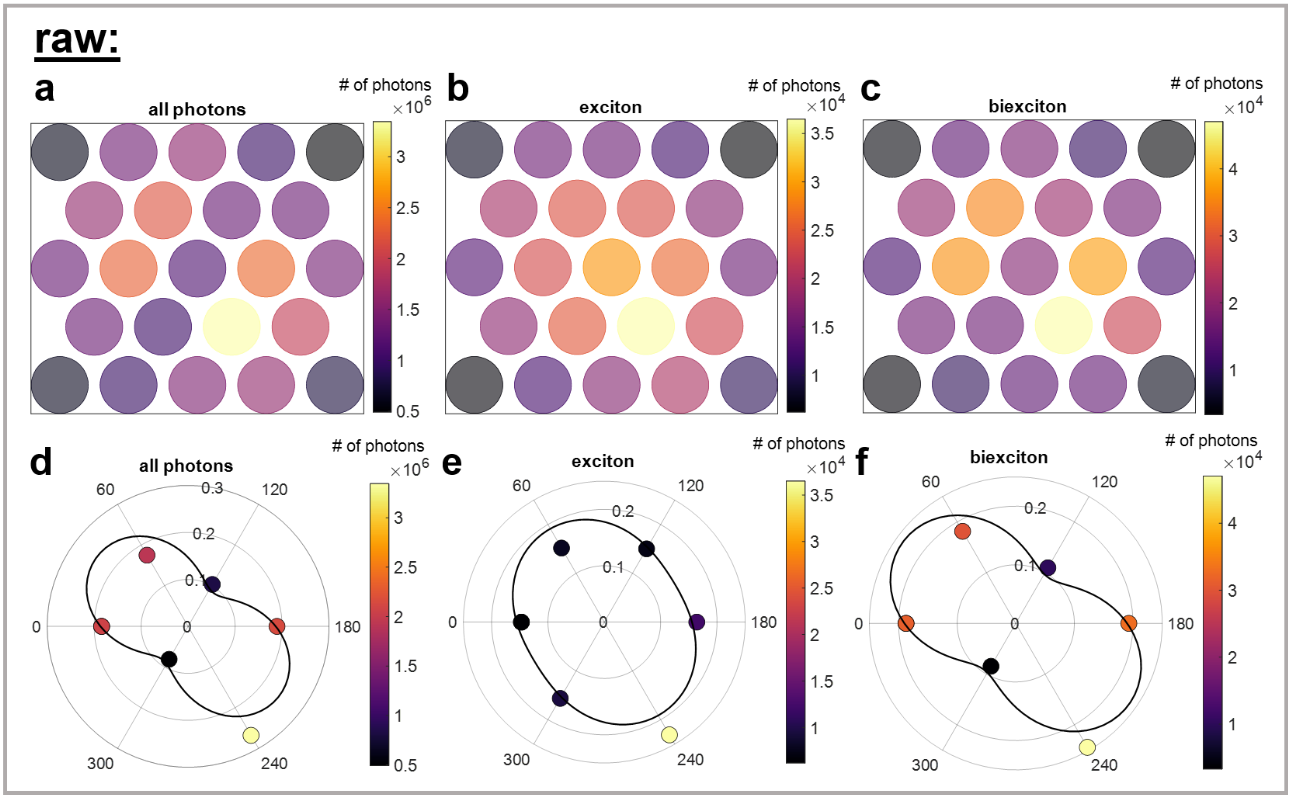
raw:
a
all photons
# of photons
b
exciton
c
biexciton
d
e
f

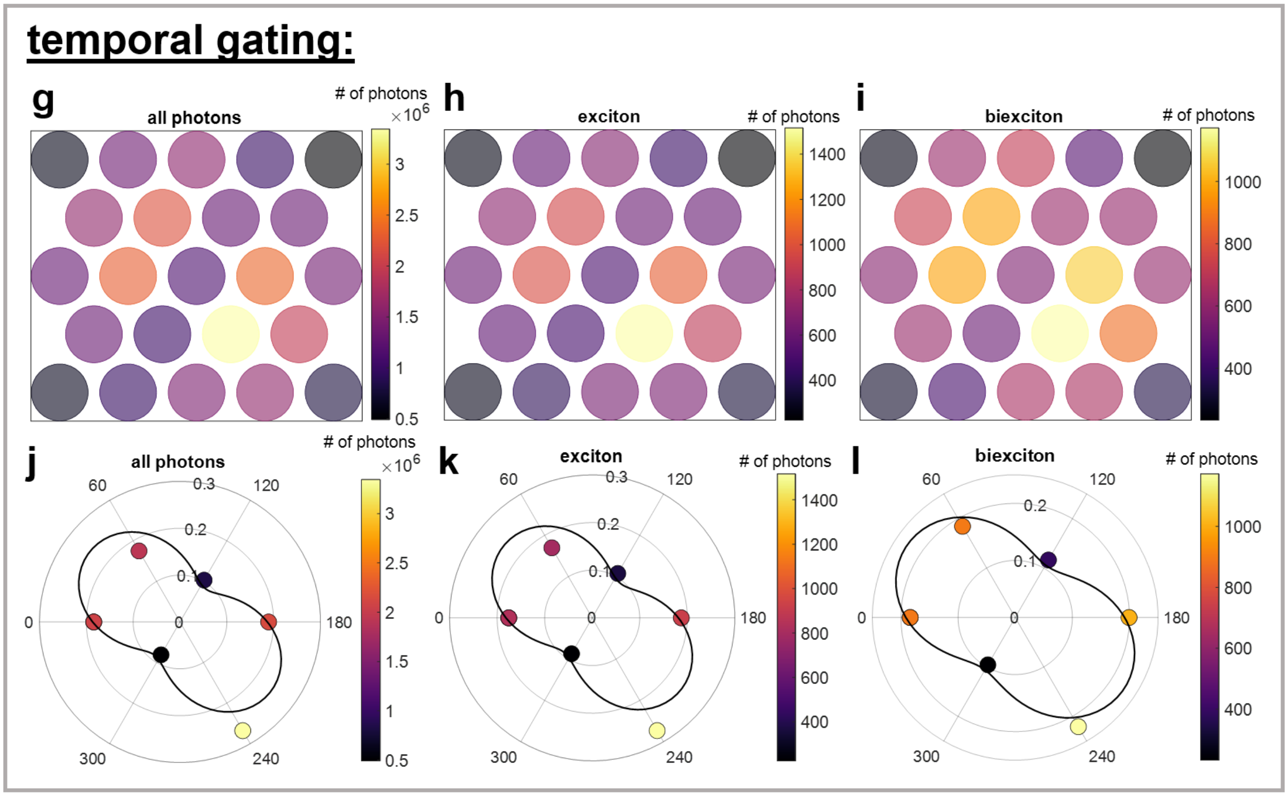
temporal gating:
g
all photons
# of photons
h
exciton
i
biexciton
j
k
l

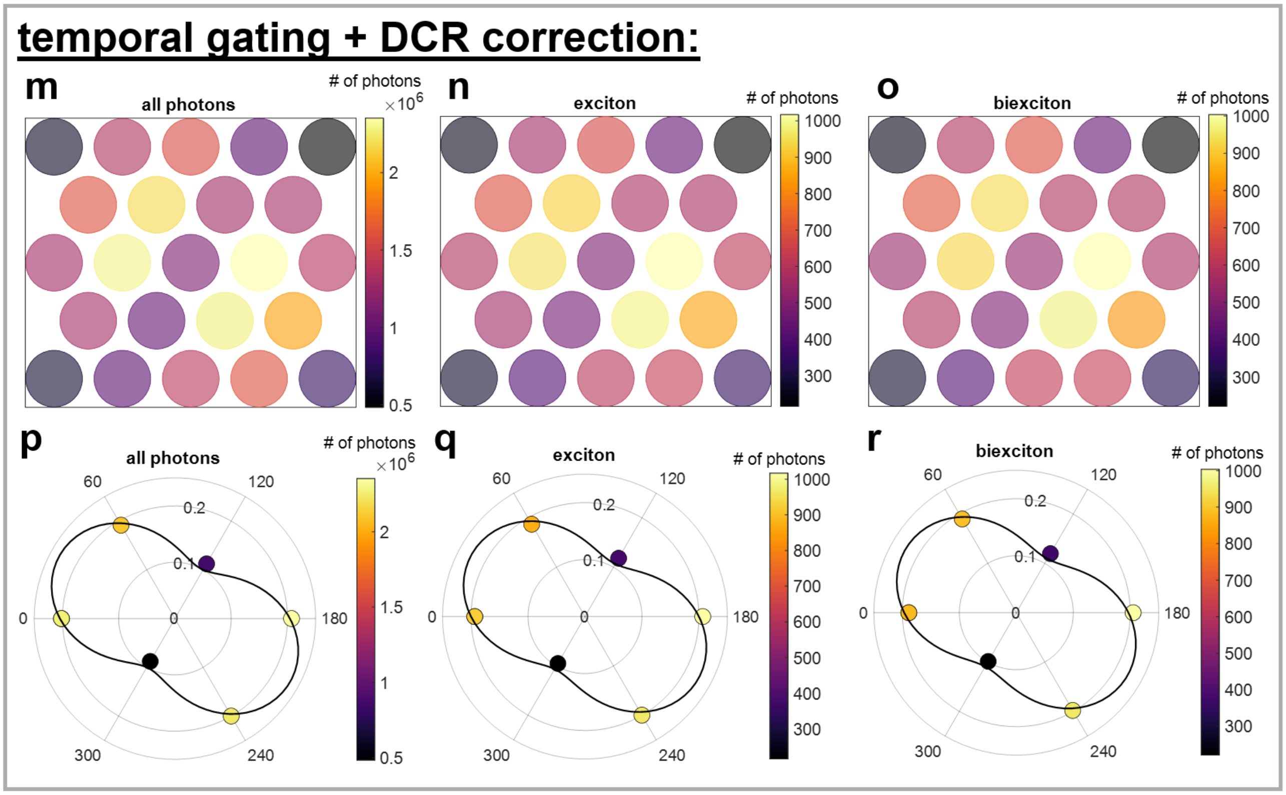
temporal gating + DCR correction:
m
all photons
# of photons
×10^6
n
exciton
o
biexciton
p
q
r
0
60
120
180
240
300
0.1
0.2

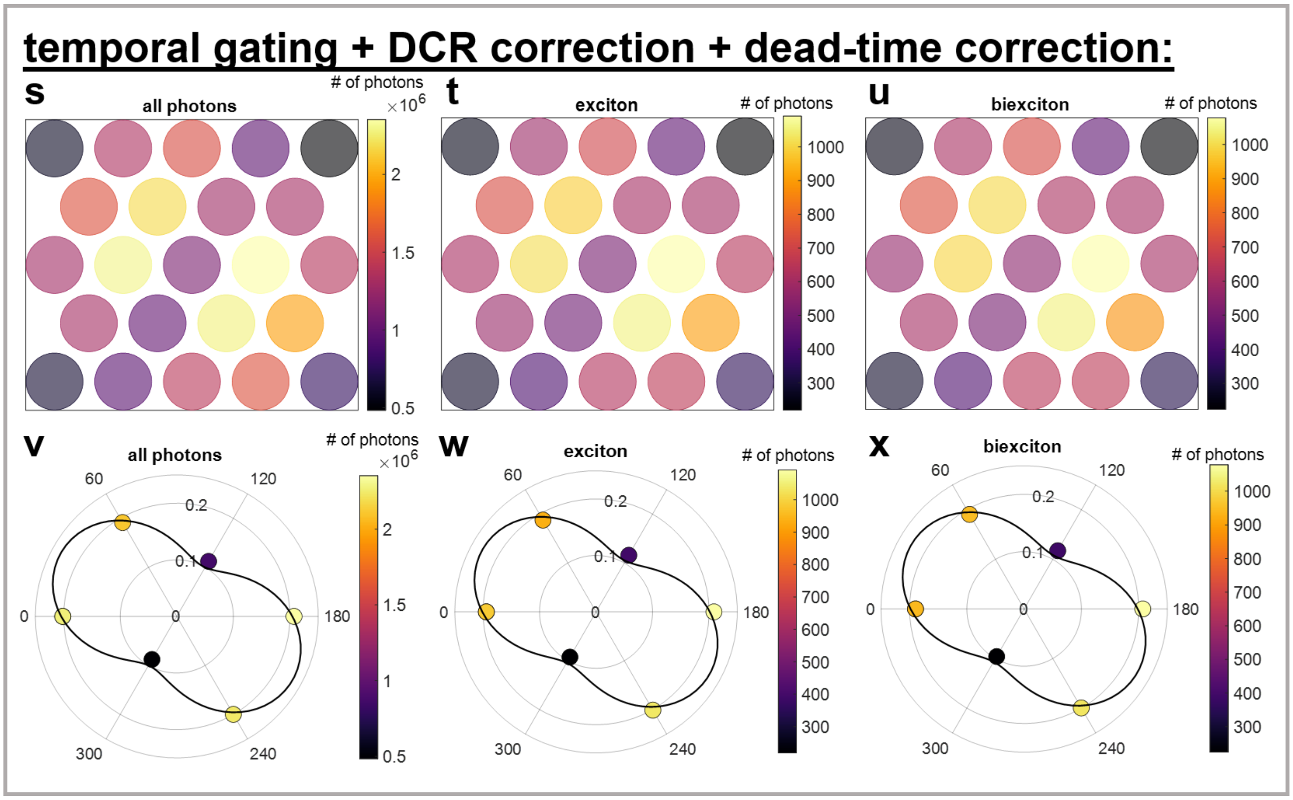
temporal gating + DCR correction + dead-time correction:
s
all photons
# of photons
×10^6
t
exciton
u
biexciton
v
w
x
0
60
120
180
240
300
0.1
0.2

Figure S5. The effect of the applied corrections on the results of a single NR, from raw data (top panel) to fully corrected data (bottom panel). (a-c,g-i,m-o,s-u) Histograms, by pixel, of all detected photons, and post-selected exciton and biexciton detections from a 5 min measurement of a single type-II ZnSe/CdS seeded nanorod (NR2, same NR presented in figure 3 of the main text), applying heralded defocused imaging. Color scale represents the number of detections at a given detector pixel. (d-f,j-l,p-r,v-x) Polar representation of the intensity values detected by the six pixels of the inner ring of the array (highlighted in figure 2b of the main text, top) for all detected photons in the measurement, along with the fit (black solid line) to the integrated dipole emission model. The six data-points are colored according to the number of detected photons in each pixel. The data presented in the top panel (a-f) is without temporal gating, DCR, and dead-time corrections. The data presented in the second panel (g-l) is after applying temporal gating of the exciton and biexciton, as explained above. The data presented in the third panel (m-r) is after applying temporal gating and DCR correction. The data presented in the fourth panel at the bottom (s-x) is after applying all the corrections mention in this section (S4): temporal gating, DCR, and dead-time, and is equivalent to the results shown in figure 3 of the main text and figure S6.

S5: Fitting to a dipole emission model for the X and BX

Figure S6 shows the results of the dipole fit applied to the X and BX emissions, as assigned by the heralded post-selection, for the NR shown in figure 3 of the main text.

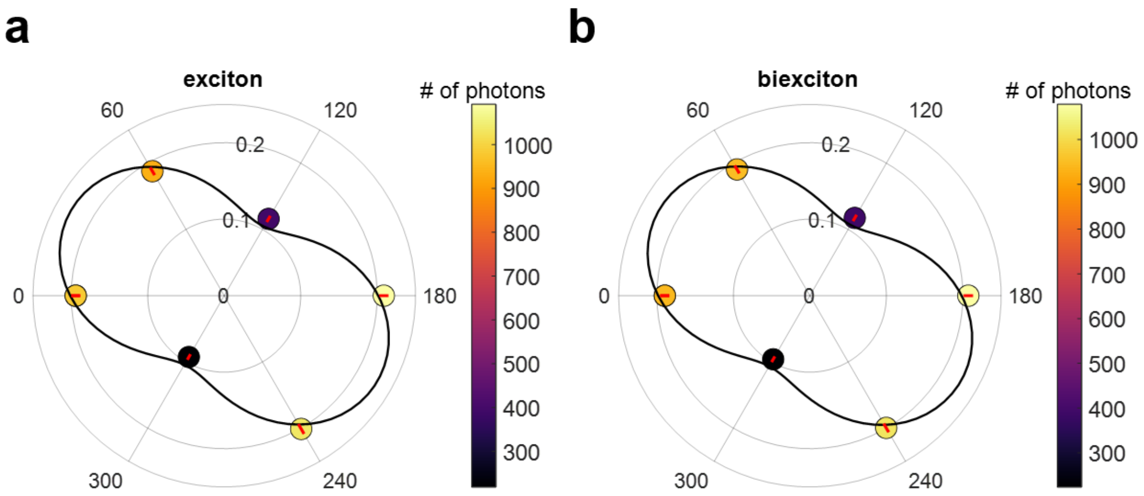

Figure S6. Polar plot of the dipole fit for the exciton and biexciton for the example shown in figure 3 of the main text. Polar representation of the intensity values detected by the six pixels from the inner ring of the array (highlighted in figure 2b of the main text) for the exciton (a) and biexciton (b) data sets along with the fit (black solid line) to the integrated dipole emission model. The six data-points are colored according to the number of detected photons in each pixel. This is complementary to the measurement presented in figure 3e of the main text. The small red lines represent the estimated experimental error due to shot noise.

S6: Heralded defocused imaging of single nanorods

Figures S7 and S8 show examples of the analysis results of single NRs (three NR1 examples and three NR2 examples, respectively) from the ensembles described in figure 4 of the main text.

Single-particle analysis for NR1

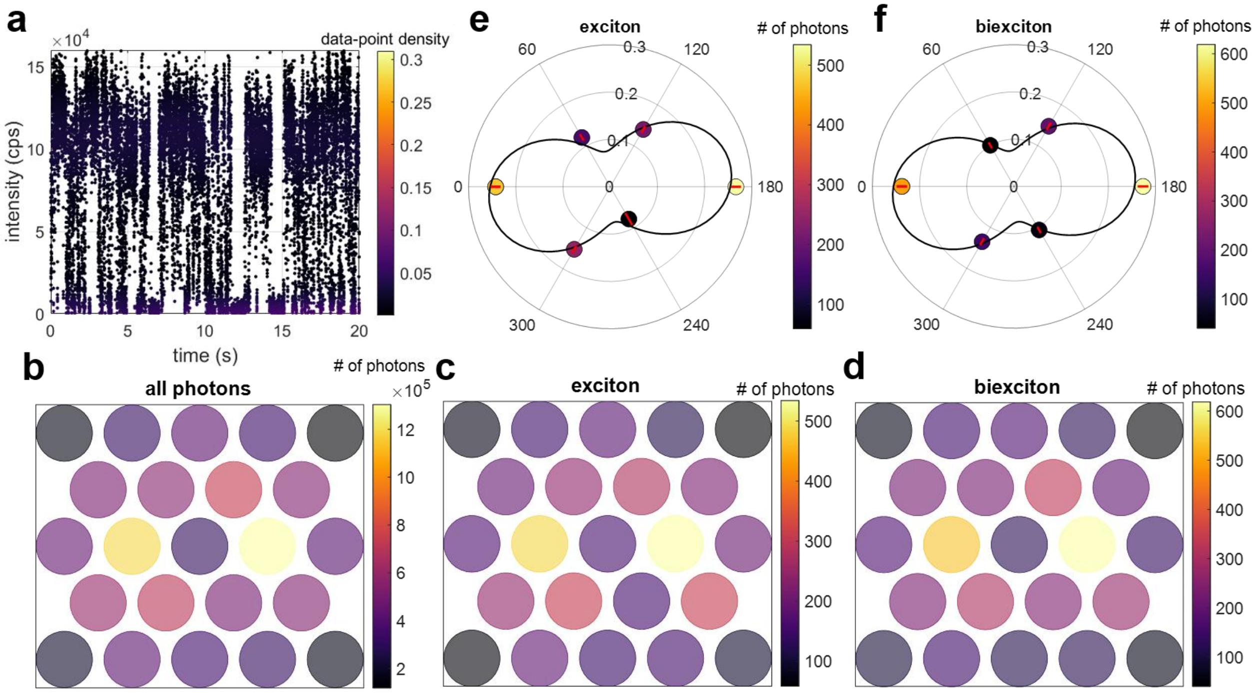

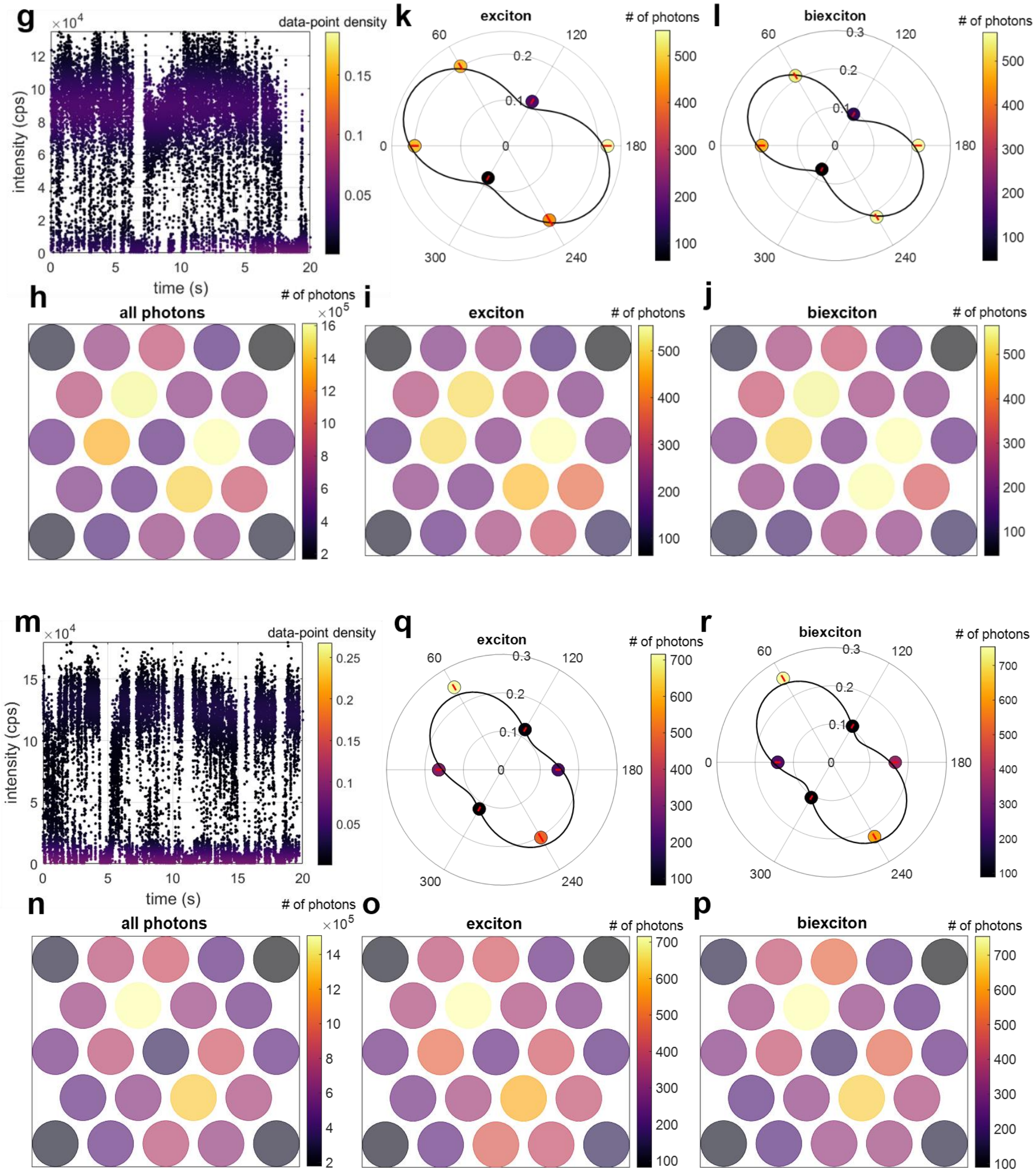

Figure S7. Three examples for heralded defocused imaging analysis of single type-I½ CdSe/CdS seeded nanorods (NR1). (a,g,m) Total fluorescence intensity collected for all pixels as a function of measurement time in a 20 second time window. (b-d,h-j,n-p) Histograms of all detected photons, titled "all photons" (b,h,n), and post-selected exciton (c,i,o) and biexciton (d,j,p) detections from a 5-minutes measurement of single type-I½ CdSe/CdS seeded nanorods (NR1), applying heralded defocused imaging. Color scale represents the number of detections at a given detector pixel. (e,f,k,l,q,r) Polar representations of the intensity values detected by the six pixels from the inner ring of the array for the exciton (e,k,q) and biexciton (f,l,r) data sets along with the fit (black solid line) to a dipole emission model. The six data-points are colored according to the number of detected photons in each pixel and the small red lines represent the

estimated experimental error due to shot noise. We note that the deviation from the model is larger than can be expected from shot noise alone. A possible source for this discrepancy may be slight lateral misalignments. Misalignments will affect the X and BX results in a similar manner, as observed in all examples in this figure.

Single-particle analysis for NR2

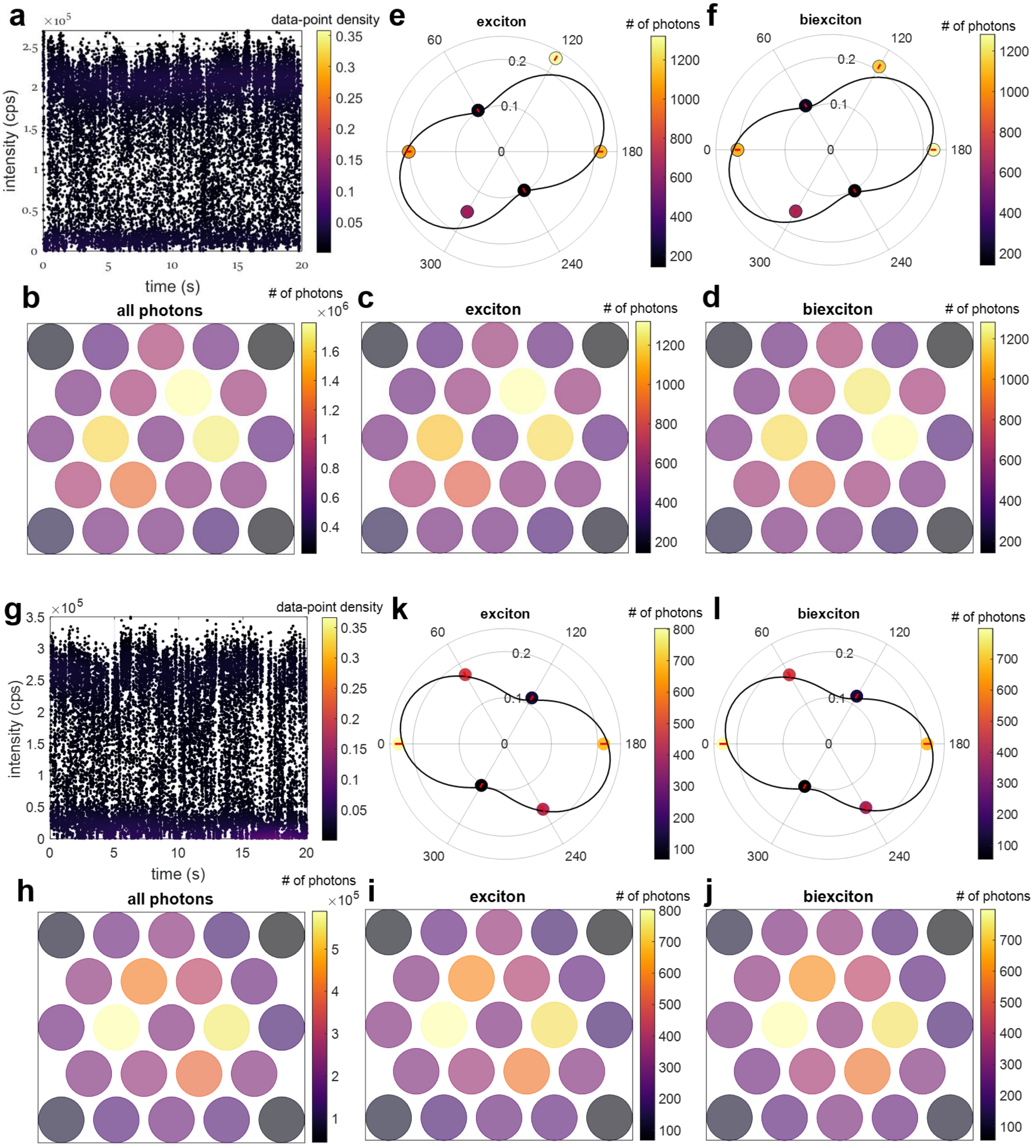

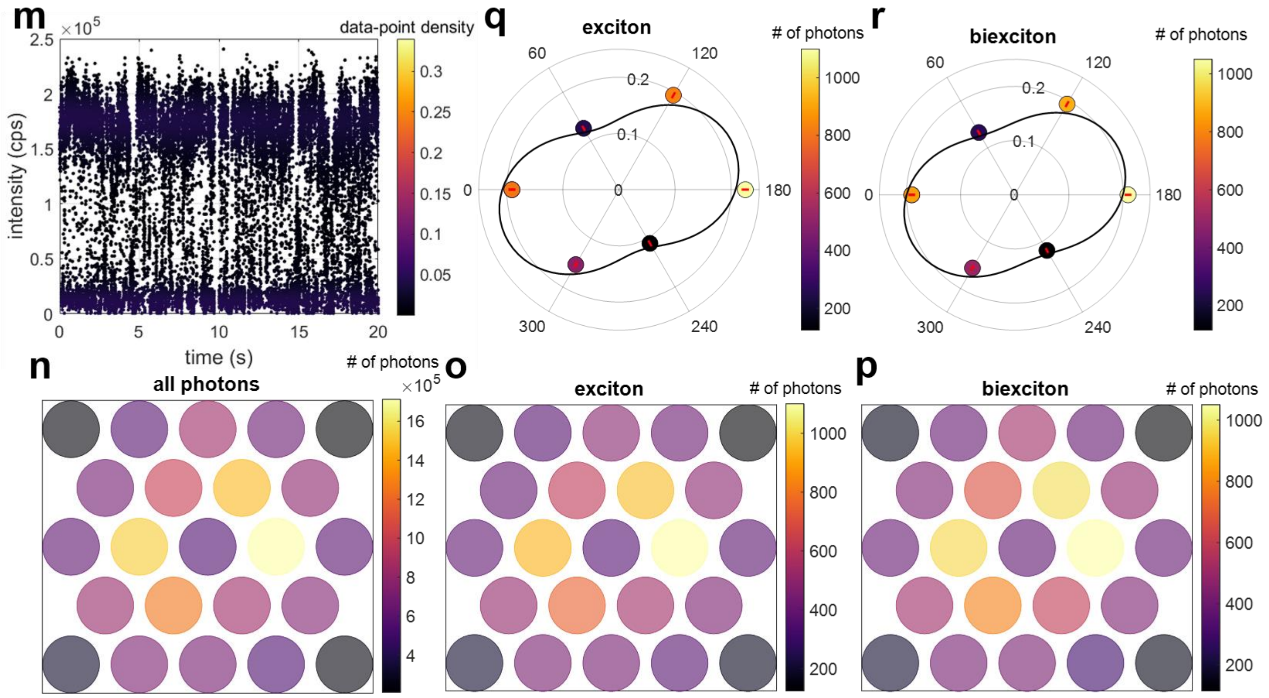

Figure S8. Three examples for heralded defocused imaging analysis of single type-II ZnSe/CdS seeded nanorods (NR2). (a,g,m) Total fluorescence intensity collected for all pixels as a function of measurement time in a 20 second time window. (b-d,h-j,n-p) Histograms of all detected photons, titled "all photons" (b,h,n), and post-selected exciton (c,i,o) and biexciton (d,j,p) detections from a 5-minutes measurement of single type-II ZnSe/CdS seeded nanorods (NR1), applying heralded defocused imaging. Color scale represents the number of detections at a given detector pixel. (e,f,k,l,q,r) Polar representations of the intensity values detected by the six pixels from the inner ring of the array for the exciton (e,k,q) and biexciton (f,l,r) data sets along with the fit (black solid line) to a dipole emission model. The six data-points are colored according to the number of detected photons in each pixel and the small red lines represent the expected shot noise error. We note that the deviation from the model is larger than can be expected from shot noise alone. A possible source for this discrepancy may be slight lateral misalignments. Misalignments will affect the X and BX results in a similar manner, as observed in all examples in this figure.

S7: Correlation of the in-plane angle of the X and BX transition dipole moments

The fitting process described in section S5 results in both the anisotropy value of the X-to-GS and BX-to-X transition dipole moments of each NR and the in-plane angle of the dipoles. As seen in figure S9, the dipole angles of the X and BX are highly correlated, as expected, which strengthens and supports the anisotropy results.

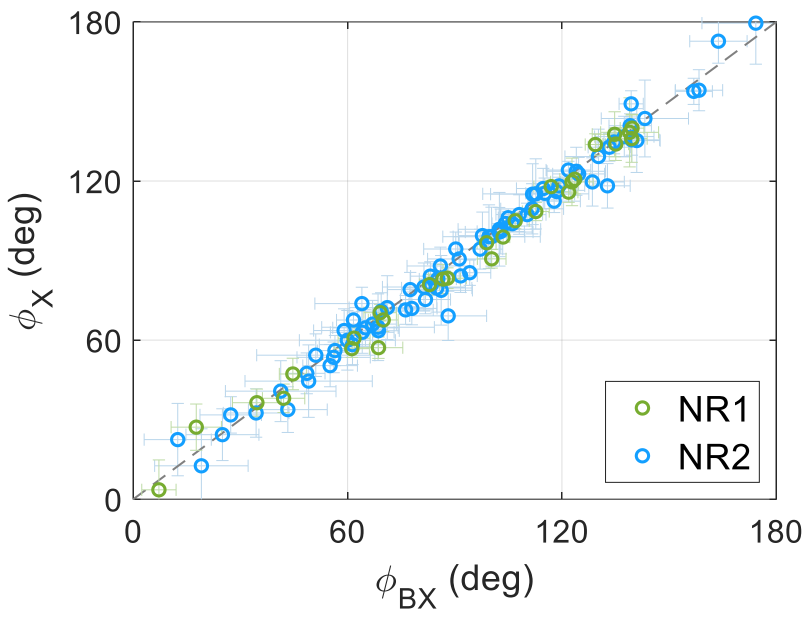

Figure S9. In-plane angle correlation. Correlation of the in-plane angle (ϕ) of the X and BX transition dipole moments for the 28 and 79 single measurements of NR1 and NR2, respectively, seen in figure 4 of the main text.

S8: Estimation of the dielectric effect

The following estimation of the dielectric effect in CdSe/CdS NRs (NR1) and ZnSe/CdS (NR2) was done according to the supporting information of ref 9. CdSe/CdS dot-in rods (similar to NR1 in this work) were approximated as homogeneous prolate ellipsoids with axes $a = b < c$, which feature anisotropic response to an excitation field. Table S1 presents the parameters needed for calculating the dielectric effect parameter, *i.e.* the ratio of the electric field strength between the major and minor axis: $R_e = \frac{f_c}{f_a}$, where f_c and f_a are called the local field factors. In our case $R_e > 1$, which implies that the attenuation of the electric field is stronger in the axes perpendicular to the rod's c-axis. Since the emission intensity scales with the square of the fields, we should take the square of R_e. ΔR_e represents the change in the dielectric effect of the CdS rod shell when the NR transitions

from being doubly excited to singly excited, followed by a small refractive index change of ~0.4% (as mentioned in the main text). α_c and α_a are the depolarization factors of the major and minor axis respectively, and they are related to the geometry of the particles.[10]

Table S1. Calculation of the change in the dielectric effect parameter. n_0 is the steady state refractive index, e is the eccentricity, α_c and α_a are the depolarization factors, and R_e is the dielectric effect parameter.

	aspect ratio	n_0 of CdS at 600 nm	e	α_c	α_a	$\lvert\Delta R_e^2\rvert / R_e^2$
NR1	5 (5x25 nm)	2.34	0.9798	0.0558	0.4721	0.96%
NR2	4 (8x32 nm)	2.34	0.9682	0.0754	0.4623	0.88%

S9: Spectroscopy of short type-II ZnSe/CdS

An additional sample of short type-II ZnSe/CdS seeded NRs (with dimensions of 8x16 nm) was measured to examine NRs with smaller aspect ratio. Figures S10a and S10b show basic characterization of the NRs, with spectra similar to the long NR2 sample shown in figure 1c of the main text. The aggregate results demonstrated in figure S10c present 23 single measurements. The diagonal is a guide to the eye, representing equal anisotropy values for the X and BX. In this correlation, all data points are distributed around the diagonal almost equally. The histogram of $\Delta_{anisotropy} = X_{anisotropy} - BX_{anisotropy}$ is demonstrated in figure S10d. Interestingly, the positive mean value suggests a trend similar to the long type-II NRs (NR2) shown in figure 4 of the main text, in which the X-to-GS transition dipole is more anisotropic than the BX-to-X transition dipole. Yet, here this observation is less significant due to lack of statistics. The statistical significance was estimated by a paired Student's t-test and yielded a score of 1 (corresponding to a p-value of ~0.3).

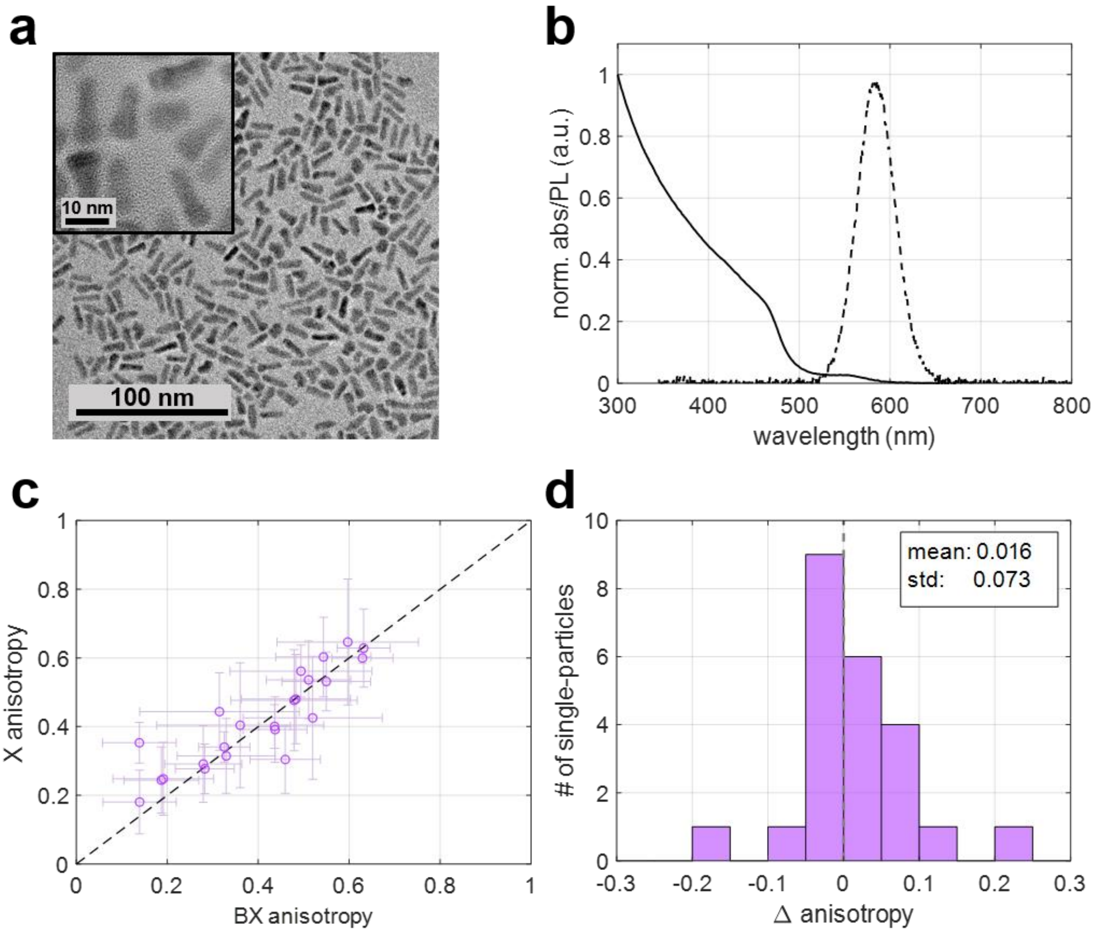

Figure S10. Characterization and heralded defocused imaging results for short type-II ZnSe/CdS seeded nanorods (NRs). (a) Transmission electron microscope image of the NRs. Inset is a high-resolution image. Scale bar is 10 nm. (b) Normalized absorbance and photoluminescence of the NRs. Emission peak is at ~583 nm. (c) Exciton anisotropy versus biexciton anisotropy values extracted from the fit. (d) Histogram of delta anisotropy values of the NRs. The dashed grey line in c and d is a guide to the eye, indicating the same anisotropy values for both photons.

1. Tyrakowski, C. M.; Shamirian, A.; Rowland, C. E.; Shen, H.; Das, A.; Schaller, R. D.; Snee, P. T. Bright type II quantum dots. *Chem Mater* **27**, 7276–7281 (2015).

2. Dorfs, D.; Salant, A.; Popov, I.; Banin, U. ZnSe quantum dots within CdS nanorods: A seeded-growth type-II system. *Small* **4**, 1319–1323 (2008).

3. Carbone, L.; Nobile, C.; De Giorgi, M.; Sala, F. D.; Morello, G.; Pompa, P.; Hytch, M.; Snoeck, E.; Fiore, A.; Franchini, I. R.; Nadasan, M.; Silvestre, A. F.; Chiodo, L.; Kudera, S.; Cingolani, R.; Krahne, R.; Manna, L. Synthesis and micrometer-scale assembly of colloidal CdSe/CdS nanorods prepared by a seeded growth approach. *Nano Lett* **7**, 2942–2950 (2007).

4. Lubin, G.; Tenne, R.; Ulku, A. C.; Antolovic, I. M.; Burri, S.; Karg, S.; Yallapragada, V. J.; Bruschini, C.; Charbon, E.; Oron, D. Heralded spectroscopy reveals exciton–exciton correlations in single colloidal quantum dots. *Nano Lett* **21**, 6756–6763 (2021).

5. Lubin, G.; Tenne, R.; Antolovic, I. M.; Charbon, E.; Bruschini, C.; Oron, D. Quantum correlation measurement with single photon avalanche diode arrays. *Opt Express* **27**, 32863–32882 (2019).

6. Lubin, G.; Yaniv, G.; Kazes, M.; Ulku, A. C.; Antolovic, I. M.; Burri, S.; Bruschini, C.; Charbon, E.; Yallapragada, V. J.; Oron, D. Resolving the controversy in biexciton binding energy of cesium lead halide perovskite nanocrystals through heralded single-particle spectroscopy. *ACS Nano* **15**, 19581–19587 (2021).

7. Rech, I.; Ingargiola, A.; Spinelli, R.; Labanca, I.; Marangoni, S.; Ghioni, M.; Cova, S. Optical crosstalk in single photon avalanche diode arrays: a new complete model. *Opt Express* **16**, 8381–8394 (2008).

8. Antolovic, I. M.; Bruschini, C.; Charbon, E. Dynamic range extension for photon counting arrays. *Opt Express* **26**, 22234–22248 (2018).

9. Vezzoli, S.; Manceau, M.; Leménager, G.; Glorieux, Q.; Giacobino, E.; Carbone, L.; De Vittorio, M.; Bramati, A. Exciton fine structure of CdSe/CdS nanocrystals determined by polarization microscopy at room temperature. *ACS Nano* **9**, 7992–8003 (2015).

10. Sitt, A.; Salant, A.; Menagen, G.; Banin, U. Highly emissive nano rod-in-rod heterostructures with strong linear polarization. *Nano Lett* **11**, 2054–2060 (2011).
